# Computational exploration of treadmilling and protrusion growth observed in fire ant rafts

**DOI:** 10.1371/journal.pcbi.1009869

**Published:** 2022-02-17

**Authors:** Robert J. Wagner, Franck J. Vernerey

**Affiliations:** University of Colorado, U.S. Mechanical Engineering Department, Material Science and Engineering Program, Boulder, Colorado, United State of America; Oxford, UNITED KINGDOM

## Abstract

Collective living systems regularly achieve cooperative emergent functions that individual organisms could not accomplish alone. The rafts of fire ants (Solenopsis invicta) are often studied in this context for their ability to create aggregated structures comprised entirely of their own bodies, including tether-like protrusions that facilitate exploration of and escape from flooded environments. While similar protrusions are observed in cytoskeletons and cellular aggregates, they are generally dependent on morphogens or external gradients leaving the isolated role of local interactions poorly understood. Here we demonstrate through an ant-inspired, agent-based numerical model how protrusions in ant rafts may emerge spontaneously due to local interactions. The model is comprised of a condensed structural network of agents that represents the monolayer of interconnected worker ants, which floats on the water and gives ant rafts their form. Experimentally, this layer perpetually contracts, which we capture through the pairwise contraction of all neighboring structural agents at a strain rate of $\dot{d}$. On top of the structural layer, we model a dispersed, on-lattice layer of motile agents that represents free ants, which walk on top of the floating network. Experimentally, these self-propelled free ants walk with some mean persistence length and speed that we capture through an ant-inspired phenomenological model. Local interactions occur between neighboring free ants within some radius of detection, *R*, and the persistence length of freely active agents is tuned through a noise parameter, *η* as introduced by the Vicsek model. Both *R* and *η* where fixed to match the experimental trajectories of free ants. Treadmilling of the raft occurs as agents transition between the structural and free layers in accordance with experimental observations. Ultimately, we demonstrate how phases of exploratory protrusion growth may be induced by increased ant activity as characterized by a dimensionless parameter, A. These results provide an example in which functional morphogenesis of a living system may emerge purely from local interactions at the constituent length scale, thereby providing a source of inspiration for the development of decentralized, autonomous active matter and swarm robotics.

## Author summary

Cooperative behavior in organisms often permits groups to achieve collective tasks that are unattainable to individuals. Such is the case of fire ant workers that aggregate together to create buoyant rafts that unify their colonies and ensure survival during floods. Under certain conditions, these rafts undergo a process called treadmilling that allows them to morph perpetually over the span of several hours. This morphogenesis includes the growth of tether-like protrusions that colonies can use as land-bridges to escape water. Employing a discrete, agent-based model, we here demonstrate how the local interactions between ants in these systems may be sufficient to cause stochastic and spontaneous protrusion emergence in the absence of external gradients, long-range interactions, or targeted stimuli. Furthermore, in isolating the effects of short-range interactions, we reveal how modulation of the activity level of freely active ants on the rafts’ surfaces may induce phase changes between exploratory states of high raft expansion, and dormant states of primarily raft contraction. These results uncover a simple set of rules by which a functional collective behavior may be achieved and may provide inspiration in the design of autonomous active systems such as swarm robotics.

## Introduction

Cooperative living systems can achieve a wide range of complex functional tasks well beyond the capabilities of the individuals that comprise them [1,2]. Perhaps chief amongst such organisms are social insects [3–5], which can operate collectively with other members of their colonies to more efficiently construct nests [6,7], thermoregulate [8,9], and forage for food [10,11]. Another fascinating example of cooperative behavior by social insects is the formation of rafts by fire ants (*Solenopsis invicta*) [12,13]. During floods, fire ants condense into buoyant rafts made entirely of worker ant bodies, thereby keeping their colonies unified and bolstering chances of survival [12,13]. Recently, we discovered and reported that rafts can maintain the ability to explore, even in flooded environments, through cooperative morphogenesis [14].

**Fig 1:**
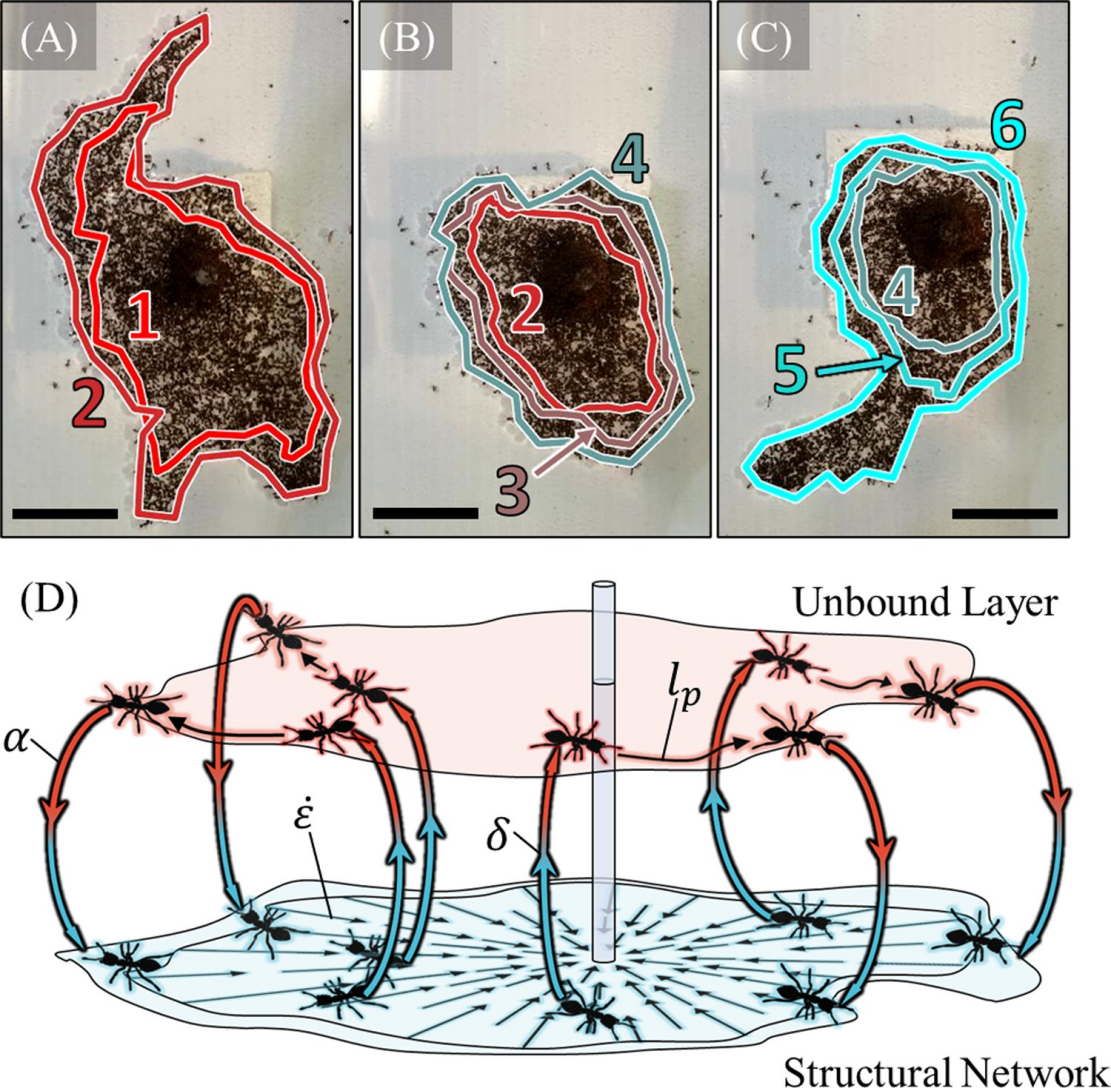
Treadmilling. **(A-C)** A top view of the same experimental raft is illustrated at the **(A)** start**, (B)** middle, and **(C)** end of a roughly 106 min duration. To visually illustrate treadmilling, a set of structural ants at the perimeter is selected every 22 minutes. These ants are then image-tracked as they flow inwards due to network contraction and the geometry defined by these ants is traced by a distinctly colored and numbered outline. The set of ants labeled “2” in **(B)**, for example, corresponds to the same set of ants labeled “2” in **(A)**, but roughly 53 min later. The label “1” represents the oldest set of ants while “6” represents the newest. The shrinking of these contours indicates retraction of the raft structure, while the existence of new layers indicates outwards expansion. Periods of raft expansion and coinciding protrusion emergence **(A,C)** were interrupted by interstitial spans of decreased activity and less eccentric morphologies **(B)**. All scale bars represent 10 ℓ where 0 mm is the approximate average body length of 1 ant. See **S1 Movie** for unannotated video. **(D)** A schematic visually illustrates the four concurrent mechanisms of treadmilling: (1) structural raft contraction at a global rate $\dot{\varepsilon }$, (2) transition of structural ants to freely active ants in the bulk at a nominal rate *δ*, (3) transport of the free ants on top of the raft with a mean persistence length *l*_*p*_, and (4) binding of free ants back into the structural network at the edges of the raft at nominal rate *α*. The schematic is taken from Wagner, et al. (2021) [14]. The freely active layer is offset from the structural layer for illustrative purposes only, as it resides directly on top of the structural network in real ant rafts. Furthermore, note that the freely active layer, while shaded continuously, is comprised of dispersed ants while the structural layer is relatively homogeneous and condensed.

In our previous work [14], we observed ant rafts containing on the order of 3,000–10,000 worker ants. When introduced to water in which a vertical rod stemmed from the surface, these ants formed dynamic raft structures comprised of a floating layer of structural ants on top of which a layer of freely active ants walked [14]. While the structural network constituted a single, condensed layer of ants with roughly conserved planar density, the freely active layer was dispersed, heterogenous and transient on the timescale of seconds. Under these conditions, these rafts display the ability to sprout tether-like protrusions that emerge and recede perpetually over the span of hours [14]. The sustained emergence of these growths relies on treadmilling dynamics in which the structural network comprising the raft continually contracts, while freely active ants on the surface of the raft deposit into the structural network’s edges and drive outwards expansion (**Fig 1**). The population of free ants that fuels outwards expansion is continually replenished by unbinding or “exit” of structural ants from the bulk of the raft and their subsequent transition into the freely active layer. We note that although the presence and dimensions of the anchoring rod may impact the behavior of fire ants in experiments, we here focus on how the experimentally measured, local behavior (e.g., trajectory properties, local interactions, *etc*.) of ants drives the treadmilling and formation of dynamic protrusions observed.

In some cases, the ants utilized protrusions as floating bridges to reach the edge of and collectively escape their containers, demonstrating that they serve an adaptive advantage. Comparable cellular systems, such as cytoskeletal walls [15–17] and cellular aggregates [18,19], display protrusion growth that, as in the case of fire ant rafts, facilitates motility and collective migration. While these cellular systems are understood to utilize chemotaxis [20], durotaxis [21] or other gradient-driven mechanisms [22] to initiate migration, it is not entirely clear whether such external stimuli are necessary to drive protrusion growth in fire ant rafts. This raises the question; do fire ants deliberately work to create these protrusions or do these features emerge spontaneously in the absence of targeted signals or external gradients? Indeed, spontaneous behaviors such as flocking of plant-animal worms [23] or ordered motion of California blackworms [24] have been demonstrated in other condensed biological systems. However, the specific circumstances and adaptive advantages, under which these behaviors occur, differ greatly from the exploratory or escape function displayed by floating fire ant rafts.

Spontaneous ordering is also well-documented in non-living active matter systems and indicates that no agent-intent is necessary to spur comparable formations [25–27]. Perhaps most similarly, Janus particles entrapped by lipid membranes have been shown to generate remarkably analogous geometries to these ant rafts due solely to stochastic, synchronous motion [28]. This occurs when a few neighboring Janus particles simultaneously apply force to the boundary that causes an acute increase in local edge curvature and runaway tether growth. Along these lines, in our previous work we treated freely active fire ants as decentralized self-propelled particles in confinement. We demonstrated that the trajectory persistence length of freely active ants is greater than the dimensions of the rafts they walk on. Under these “strongly confined” conditions, it is known that self-propelled particles cluster near the convex edges of their containment geometries [25,29]. Employing a coarse-grained continuum model of ants on protrusions based on the work of Fily, et al. (2014) [25], we postulated a mechanism through which local breaks in convex symmetry at the rafts’ edges may induce a runaway feedback loop whereby the locally higher curvature causes clustering of free ants, and the higher concentration of free ants causes a higher local edge deposition rate. Yet, we also demonstrated that a higher concentration of free ants alone does not lead to the elongated morphologies observed in the experimental system and there must exists some mechanism that biases the direction in which ants bind into the structural network [14]. While the hypothesized source of this bias is a first or second-order effect of the directional motion of free ants on protrusions [14], its true origins remain unclear and whether such protrusion may form in ant rafts due solely to local interaction rules alone is uncertain. However, further exploration through the continuum model is limited by smoothing assumptions that prohibit the investigation of phenomena such as individual ant behavior, heterogeneities, and discrete size effects. Additionally, exploration of this bias through experimentation is limited by factors such as small sample sizes (since protrusions must be allowed to occur spontaneously without interference), difficulty in image-tracking the position of free ants, and the inability to measure variables such as ant self-propulsion force. For these reasons, a discrete modeling approach such as that taken by Vutukuri, et al. (2020) [28] or other researchers in the study of ant species [30–35] is warranted.

We here develop and employ a 2D, ant-inspired, agent-based, numerical model in which the behavior of every single ant in the structural and freely active layer is discretely captured. In matching the statistical behavior of agents to ants in both layers, we use this model to demonstrate that a set of local interaction rules predicts the emergence of spontaneous protrusion growth in the absence of any long-range communication or external gradients. These rules confirm that biased motion of free agents occurs on protrusions given the condition of strong confinement and local alignment interactions, and this directional motion facilitates the runaway growth of said protrusions, as hypothesized previously [14]. Furthermore, we use this model to investigate another unexplored phenomenon: oscillatory phase changes in fire ant rafts between highly eccentric periods of outwards expansion accompanied by protrusion growth, and recessive periods in which the rafts assume more rounded or elliptical shapes (**Fig 1A–1C**). Comparable cyclical changes in the mechanical properties of 3D aggregations of fire ants have been documented and attributed to shifts in the activity level of the overall population [36–38]. We find here that morphological phases of ant rafts may similarly be modulated through the activity level of freely active ants on their surface as characterized by a dimensionless activity parameter A. This parameter represents the competition between ants’ self-propulsion force, and their effective repulsion from the raft edge due to their dislike of water. In the remainder of this work, we introduce the model and confirm that it replicates the treadmilling dynamics observed experimentally. We then demonstrate that it predicts the spontaneous formation and runaway growth of protrusions, despite initially circular raft geometries. Finally, we explore how modulating activity induces phase transitions between periods of outwards, exploratory growth, and contractile withdrawal, as seen in experiments.

## Results

### Modeling ant rafts

**Fig 2:**
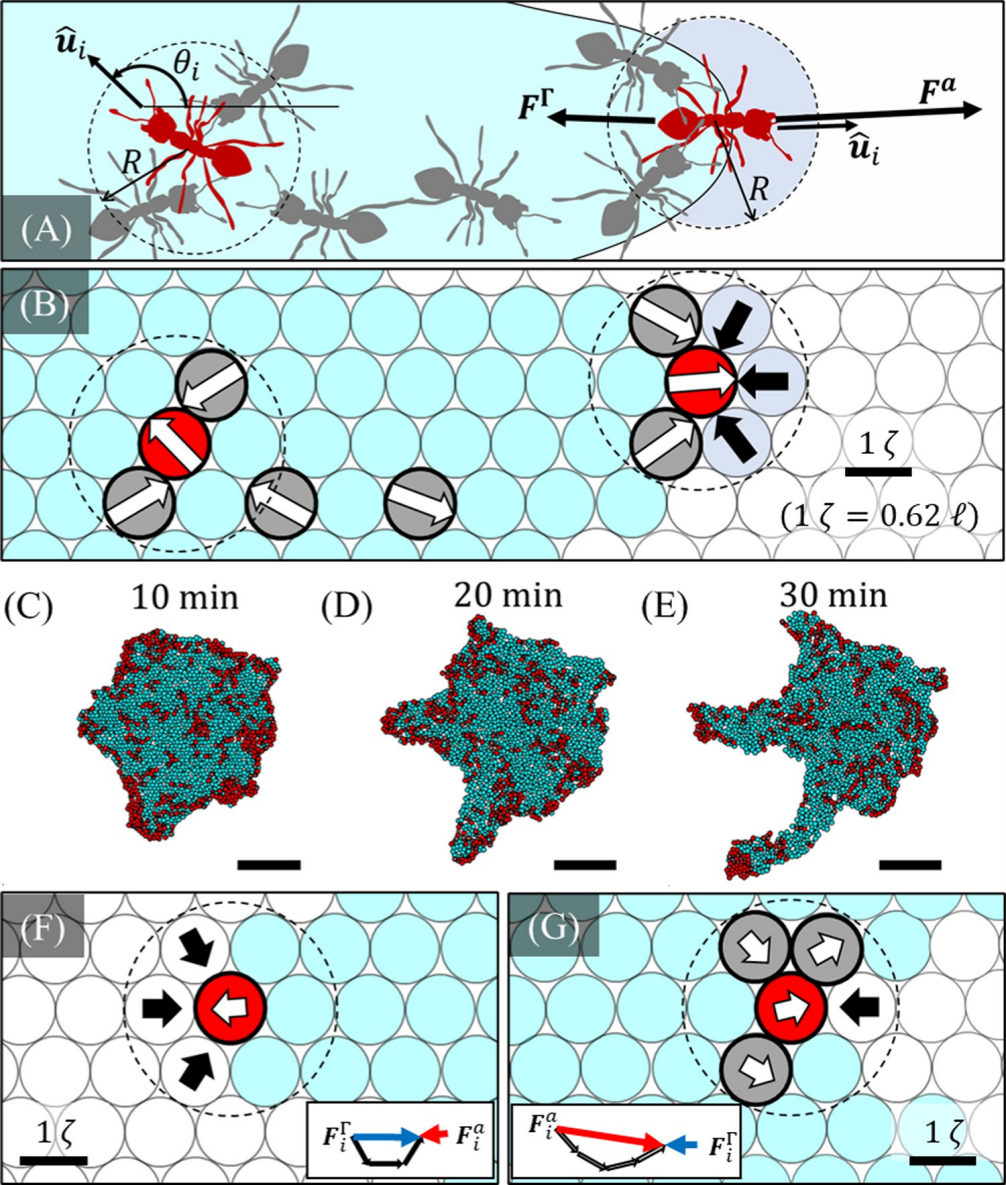
Agent-based Model Schematic. **(A)** Two free ants of interest (red) are schematically illustrated on a structural section of raft (shaded cyan) in continuous space. Other free ants are shaded grey. The direction of motion (${\hat{u}}_{i}=[\cos\theta_{i},\sin\theta_{i}]$) of the ant far from the edge of the raft (left) is predicted entirely by the Vicsek model. In contrast, whether the ant encountering the edge of the raft (right) moves into the water, depends not only on ${\hat{u}}_{i}$, but also on the competition between active force $F_{i}^{a}$ and the effective edge repulsion force $F_{i}^{\Gamma }$. Each of these forces is governed by the motion of free ants and relative position of water within detection distance *R* of the ants. **(B)** A corresponding schematic envisions how these continuous scenarios are coarse-grained into the lattice-based framework of the numerical model. The motion of the free agents of interest (red) remains governed by the direction of travel (white arrows) of neighboring free agents, and effective pairwise repulsion (black arrows) from neighboring water nodes within distance *R*. However, free agent movement is updated by stepping the free agents to the adjacent structural agents (cyan) or water nodes (white) whose relative direction most closely matches the preferred direction, *θ*_*i*_. Nodes are displayed in a hexagonal, close-packed lattice for illustrative purposes only, but are initially offset in both directions of the horizontal plane by some amount in the range*ζ* and are further randomized by stochastic structural unbinding events as the simulation progresses. **(C-E)** The shape evolution of a simulated raft over a duration of 20 min (of virtual time), illustrates the implementation of the lattice-based conceptualization from **(B)** into the numerical model. Shape change is governed by the transition of free agents (red) into the structural network (cyan) at the raft’s edge. The raft depicted was initiated as a circle and all scale bars represent ℓ. (**F-G**) Agents encountering water in regions of (**F**) high and (**G**) low edge curvature are depicted. These respective agents experience high and low values of ***F***^Γ^ due to the pairwise contributions of repulsion force from detected water nodes (black arrows). The agent in (**F**) has no freely active neighbors such that the only contribution to its value of ***F***^*a*^ is its own self-propulsion force (white arrow), whereas the agent in (**G**) has many freely active neighbors moving in similar directions towards the water such that it has a high value of ***F***^*a*^ oriented off the raft. **(F-G)** Insets display the vectorial sums that define the effective forces ***F***^*a*^ (red) and ***F***^Γ^ (blue) for the respective agent configurations, thus illustrating how the agent in (**G**) is more likely to edge-deposit based on **Inequality 2**.

Here we overview the discrete numerical model used in this work to contextualize the results presented. Detailed derivations and implementation methods are provided, as needed, in the **Materials and Methods** section. We see in our previous work that treadmilling of ant rafts is driven by four concurring mechanisms: (1) perpetual contraction of the floating, structural ant network, (2) exit of structural ants out of the network into the freely active layer, (3) self-propulsion of the free ants on top of the raft, and (4) deposition of free ants into the structural layer at the raft’s edges (**Fig 1D**) [14]. To capture these mechanisms, the model represents ants as discrete agents whose motions are confined to a lattice of water nodes. To represent the two-state nature of ant rafts, the model consists of a population of structural agents representing the raft’s structural network, on top of which a population of freely active agents moves dispersedly. These respective populations are denoted by the colors cyan and red throughout this text unless specified otherwise. The positions of structural agents and water nodes are updated in continuous space to capture the mechanism of structural network contraction. However, the movement of free agents is constrained to the lattice defined by the structural agents, thus naturally ensuring that free agents can only occupy the spatial domain of the raft. An illustrative schematic of two hypothetical free ants in continuous space is depicted in **Fig 2A**, while the corresponding conception of free agents in the lattice-based model is shown in **Fig 2B**. Although the respective states of structural and free ants may consist of multiple layers distributed in the *z-*axis (depending on the time of inspection) [12,14], we here choose to model each as a single layer of agents based on the experimental observation that during phases of protrusion growth, the structural network generally spread into a monolayer (with a planar density of 0.304 ants mm^−1^) and the freely active layer was–on average–dispersed with a mean packing fraction of approximately 0.24 free ants per structural ant [14]. **Fig 2C–2E** depicts snapshots of a simulated raft in which the monolayered structural network is represented by cyan lattice sites, and the dispersed active layer on top is depicted by red free agents. While these two states behave independently in the model, agents transition between them according to a set of ant-inspired rules.

### Modeling structural agents

**Fig 3:**
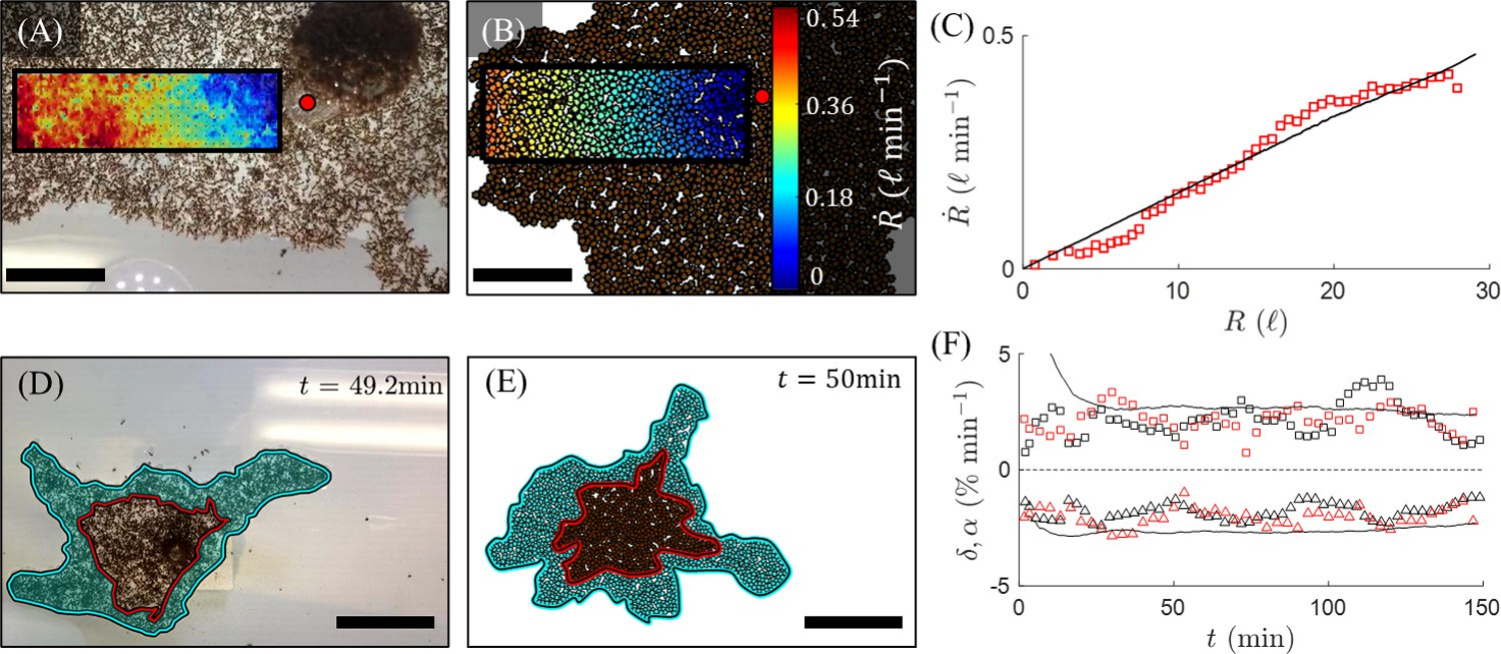
Comparing Treadmilling Dynamics. **(A-B)** The gradient of contractile speed, $\dot{r}$, towards the anchor point of the rafts (red dot) is illustrated via heat maps within defined regions of interest (ROIs) for both **(A)** an experimental and **(B)** simulated raft. $\dot{r}$ was computed as the component of speed moving towards the stationary reference frame (i.e., the acrylic rod) and was measured for every point within these 2D ROIs, then averaged over durations exceeding 13 minutes. Scale bars represent 10 ℓ. **(C)** $\dot{r}$ is plotted with respect to distance from the anchor point, *r*, for both the experiment (discrete red squares) and simulation (solid black curve). The slopes of the least-squares regression lines are taken as the average contractile strain rate $\dot{\varepsilon }$. The experimental strain rate ($\dot{\varepsilon }=1.63\pm 0.01\;\%\;{min}^{-1},R^{2}=0.96$) agrees with the numerical value ($\dot{\varepsilon }=1.62\;\%\;{min}^{-1},R^{2}=0.99$). **(D-E)** The growth zones of both **(D)** an experimental and **(E)** simulated raft after roughly 50 min are shaded in cyan. Scale bars represent 15 ℓ. The bound ants that occupied the perimeter of the raft at reference time, *t*_0_ = 0, are outlined in red and were traced through time. **(F)** The time-evolution of the edge binding rate, *α*, and bulk unbinding rate, *δ*, as a percentage per unit raft area are shown for two sets of experiments (squares for *α* and triangles for *δ*; red and black for two different experiments) along with the averaged results of 12 simulations (continuous black curves). Note that the initial drops in both *α* and *δ* for the simulation data occur since the raft was not initiated at steady state, whereas experimental data was only sampled at pseudo-steady state. **(A,C,D,F)** Experimental results are courtesy of Wagner, et al. (2021) [14]. All simulated rafts were initiated as circles and shape was allowed to evolve stochastically.

Based on experimental evidence we find that raft contraction is relatively constant in time [14]. In contrast the deposition of free ants that drives outwards raft expansion varies significantly over hours, with some free ants clustering near the rod in an inactive state. Therefore, in the scope of this work, our primary aim is to explore the local, free agent behavior that drives phase changes in these systems. Naturally, the model must still replicate the global treadmilling dynamics that are prerequisite to sustained shape change and for which global contraction is an essential mechanism. We found previously that the structural layer contracts uniformly throughout the network and that its density is roughly conserved even over long time frames [14]. To capture this uniform global contraction without introducing mechanisms that would require long-range cooperation, we introduce spatially continuous pairwise contraction between neighboring structural agents at a constant strain rate of $\dot{d}\;[\%\;{min}^{-1}]$. It remains unclear if structural ants contract towards all of their nearest neighbors or if they only contract to fill in voids originating at sites where ants recently exited the structural layer. Regardless, that global contraction is observed mandates that there exists microstructural contraction at some length scale, which we here enforce between all pairwise nearest neighbors for simplicity. This ensures that the mechanisms driving global contraction could feasibly be achieved by agents working through exclusively local interactions. Setting $\dot{d}$ to 1.2 times the globally measured contractile strain rate ($\dot{\varepsilon }$) led to good agreement between experiments and simulations (**Fig 3A–3C**) [14] (see the **Materials and Methods** section **Simulating the Structural Network** for details). The fact that $\dot{d}$ does not equal $\dot{\varepsilon }$ generally indicates that the local rate of contraction between nearest neighbors is higher than the emergent global rate, which is expected in a network due to non-affine effects [39].

To avoid hindering contraction, volume exclusion between structural agents is not enforced. However, unconstrained network contraction would lead to a ceaseless increase in structural ant concentration, which was not observed experimentally [14]. To ensure conserved planar network density structural agents are unbound and converted to freely active agents wherever their local quantity per unit area (i.e., their density) exceeds a prescribed threshold. This allows for robust exit of structural ants throughout the bulk, as observed in experiments [14]. To match experiments, this threshold was set to 1 agent per *ζ*^2^, where *ζ*^2^ is the area occupied by one experimental, structural ant ($\zeta =\rho_{r}^{-0.5}=1.81\pm 0.30$ mm, where *ρ*_*r*_ is the planar structural network density). Consequently, a numerical rate of structural unbinding, min^−1^, naturally emerged and matched experimental estimates (**Fig 3F**) [14], suggesting that 2% of structural agents convert to freely active agents every minute. Thus, through this pairwise contraction, both global network contraction and flux of ants from the structural network to the freely active layer were achieved. Note that the structural agents provide a continuously updated lattice on which the freely active agents move, such that structural layer contraction also induces contraction of the free layer. However, active agents walk at speeds two orders of magnitude greater than that of the structural contraction such that the effect of contraction is negligible on free agents.

### Modeling freely active agents

While global contraction and bulk structural unbinding are essential in replenishing the population of freely active ants, it is ultimately the deposition of these free ants into the edge of the structural network that governs global shape evolution. This deposition is largely dependent on the distribution of free ants at the edge, and therefore the transport of free ants on the surface of the raft. To model surface traffic, we begin with the qualitative observations that free ants are self-propelled agents whose trajectories under weak confinement are isotropic but correlated below the length scale of one ant body length (1 ℓ), indicating some degree of local alignment interactions [14]. To capture this local alignment, the phenomenological Vicsek model [26] is used to predict the preferred direction of motion of self-propelled agents as they traverse the structural lattice. Through this model, the preferred angle of motion of free agent *i* at time *t*+Δ*t* is updated according to [40]:

$$\theta_{i}(t+\Delta t)={\left\langle \theta_{j}(t)\right\rangle }_{i}+\xi_{i}(t),$$ (1)

where ⟨*θ*_*j*_(*t*)⟩_*i*_ is the average orientation, *θ*, of all neighboring freely active agents (including agent *i*) at time *t*. Neighboring agents are denoted by the index *j* and defined as free agents residing within some detection distance, *R*, of agent *i*. Generally, throughout this work the indices, *i* and *j* denote the agent of interest and its influencing neighbors, respectively, rather indicating vector values. Vectors are instead denoted by bold text. Similarly, where used, combined indices (e.g., “*ij*”) denote pairwise values or values from *j* to *i*, rather than second order tensors. Note that raft agents exert no influence on active force since they reside beneath the plane of active agents and move at considerably slower speeds. The scalar value *ξ*_*i*_ induces a random change in agents’ directions within the uniformly distributed range [−*πη*, *πη*], thus coarsely capturing decision-based noise in ants’ trajectories. Here *η*∈[0,1] is the noise parameter introduced by Vicsek, et al. (1995) [26] (**Fig 2A**), whereby if *η* = 0 there is no noise and if *η* = 1 the movement of agents is completely random. The Vicsek model is a minimalist model for active particle motion that may capture a full spectrum of phases from fully isotropic motion (at low densities or high noise) to completely uniform movement (at high densities or low noise), and which depends on just three parameters: particle density, the radius of influence (*R*) and noise (*η*) [26]. Therefore, it is favored for its versatility, simplicity and is applied here given the experimental evidence that there exists mutual interactions between free ants, albeit only at the contact length scale [14]. Although this model is commonly used to capture collective motion driven by non-local interactions between self-propelled particles, it is not exclusive to collision-avoiding particles, and we here set *R* such that only neighbors in contact may directly influence one another. In this lattice-based framework, *η* and *R* are taken as 0.2 and 0.9 ℓ, respectively, in order to replicate the experimentally measured persistence length, *l*_*p*_, of free ant trajectories far from rafts’ edges [26] (see the **Materials and Methods** section **Vicsek Model Parameters** for details).

At every timestep, **Eq 1** is used to define the preferred direction of motion for each free agent. However, since free agents are constrained to the lattice of structural agents, **Eq 1** is not used to step their positions continuously. Instead, each agent is assigned (at most) 18 movement degrees of freedom (DOF) that may consist of either structural agents or water nodes, but which must exist within some distance *R*_*DOF*_ of agent *i* (see the **Materials and Methods** section **Stepping Free Agent Movement** for selection of *R*_*DOF*_). Note that *R*_*DOF*_ naturally constrains the maximum speed of free agents to *v*_*max*_ = *R*_*DOF*_/Δ*t*, and defines some mean distance, ⟨*d*⟩, between agent *i* and its neighboring DOF. Indeed, this permits that the time step be set to according to Δ*t*≈⟨*d*⟩/*v*_0_, where *v*_0_ is the experimentally measured mean free ant speed, thereby calibrating the timescale of this model. Once *θ*_*i*_ is computed, agent *i* is then stepped to the position of the “eligible” DOF whose relative orientation most closely matches *θ*_*i*_ (see the **Materials and Methods** section **Stepping Free Agent Movement** for details). Eligibility is defined by a set of ant-inspired criteria. Firstly, structural DOF are ineligible if they are already occupied by a free agent. This mimics volume exclusion interactions between free ants (i.e., that two ants cannot occupy the same space) and naturally enforces that the freely active agents occupy a monolayer and remain relatively dispersed as in experiments [14]. Secondly, eligible DOF must exist within the confines of some turning limit (±*π*/2 radians) with respect to *θ*_*i*_. This turning limit was included due to the observation that it takes free ants greater than Δ*t* to turn more than approximately *π*/2 radians, thereby limiting the turning rate of agents using an approach similar to that of Couzin and Franks (2003) in their investigation of army ants (*Eciton burchelli*) [35]. Finally, if the preferred DOF is a water node then an additional rule of edge deposition must be satisfied to allow movement, as discussed in the following section. If a free agent has no eligible movement DOF, it pauses before re-evaluating its preferred direction of motion according to the algorithm described in the **Materials and Methods** section **Pausing Surface Traffic**.

### The rule of edge deposition

To properly model edge deposition, we again begin with experimental observations. Active ants appear to “encounter” the raft’s edge when they walk directly towards it and contact the water. These ants avoid binding into the raft’s edge (which requires moving into the water) unless pressured by neighboring active ants and adequately surrounded by structural ants upon deposition into the network. Together, these observations indicate competition between some effective active force $F_{i}^{a}$ due to a free ant’s self-propulsion combined with that of its nearest neighbors, and some effective edge repulsion force $F_{i}^{\Gamma }$ that occurs at the perimeter of the raft. Here, $F_{i}^{\Gamma }$ is not a physical force, but rather an embodiment of ants’ motivation to stay on dry substrates and is akin to the "social forces" employed by Helbing and Molnár (1995) [41]. Whether or not it is due to free ants’ affinity to the raft, aversion to water, or both is not immediately clear or relevant. Nevertheless, there is a clear observational tendency for individual ants to avoid moving into the water under their own volition.

To mimic these experimental observations, we define an edge encounter as occurring when a free agents’ preferred movement DOF is a water node. We then compute $F_{i}^{a}$ and $F_{i}^{\Gamma }$, and simply define an edge deposition event as occurring when the magnitude of active force driving the agent off the raft is greater than the magnitude of effective edge force repelling it from the water, in the direction of agent motion (${\hat{u}}_{i}=[\cos\theta_{i},\sin\theta_{i}]$). Mathematically, this is given by:

$$(F_{i}^{a}+F_{i}^{\Gamma })\cdot {\hat{u}}_{i}>0.$$ (2)

To compute $F_{i}^{a}$ we coarsely assume that self-propulsion forces are fully transmitted between in-contact free agents such that:

$$F_{i}^{a}=Nf^{a}\varphi_{i}^{\sigma },$$ (3)

where *f*^*a*^ is the magnitude of a single agent’s self-propulsion force and *N* is the number of neighboring free agents (*σ*∈[1,*N*], inclusive of *i*) residing within the contact radius *R*. Here, $\varphi_{i}^{\sigma }$ is the local order vector in free agent motion defined by $\varphi_{i}^{\sigma }=N^{-1}\sum_{\sigma }^{N}{\hat{u}}_{\sigma }$ where ${\hat{u}}_{\sigma }$ is the direction of motion (${\hat{u}}_{\sigma }=[\cos\theta_{\sigma },\sin\theta_{\sigma }]$) of neighboring free agent *σ*. The local order vector is **1** when all local free agents are moving in the same direction, and approaches **0** when the local movement is completely disordered [42]. Therefore, $F_{i}^{a}$ scales with the degree of local synchronization (or cooperation) between free agent motion through $\varphi_{i}^{a}$, and is bound by the number of locally detected neighbors, *N*.

To compute ***F***^Γ^ we consider that there exists some effective pairwise repulsive force, $f_{i\omega }^{\Gamma }$, acting on agent *i* due to each of its *N* adjacent water neighbors (*ω*∈[1,*N*]) within distance *R*. Treating this force as the negative gradient in potential energy between the positions of node *ω* and agent *i* (i.e., $f_{i\omega }^{\Gamma }=-\nabla_{r}U$), assuming the simplest case of a linear energy gradient between these sites, and recognizing that detection distance *R* is the contact length scale (i.e., *R*/ℓ~1) then we may take the magnitude of $f_{i\omega }^{\Gamma }$ as a constant, *f*^Γ^ (see the **Materials and Methods** section **Effective Edge Repulsion** for details). Assuming a linear superposition of the pairwise forces then the net repulsive force on *i* may be computed as the sum of discrete contributions [43] from all *N* water neighbors as:

$$F_{i}^{\Gamma }=-Nf^{\Gamma }\varphi_{i}^{\omega },$$ (4)

where $\varphi_{i}^{\omega }$ is a local order vector ($\varphi_{i}^{\omega }\in [0,1]$) indicating the relative position of water nodes with respect to *i*. Mathematically, $\varphi_{i}^{\omega }=N^{-1}\sum_{\omega }^{N}{\hat{r}}_{i\omega }$ where ${\hat{r}}_{i\omega }=(X_{\omega }-X_{i})/|X_{\omega }-X_{i}|$ is the direction vector from the position of *i* (***X***_*i*_) to the position of *ω* (***X***_*ω*_), which points towards the average location of detected water nodes. The magnitude of $\varphi_{i}^{\omega }$ increases as the relative orientation of these water nodes becomes more tightly grouped with respect to *i* (e.g., if all detected water nodes are approximately in-line with and on one side of *i*, then $\varphi_{i}^{\omega }\to 1$). As a result, $F_{i}^{\Gamma }$ scales proportionately to the amount of water detected through *N* and acts approximately in the direction normal to (and inwards from) the raft’s boundary through $-\varphi_{i}^{\omega }$.

Substituting **Eqs 3** and **4** into **Inequality 2** provides a normalized condition for edge deposition:

$$\frac{f^{a}}{f^{\Gamma }}\left(\frac{N^{\sigma }\varphi^{\sigma }}{N^{\omega }\varphi^{\omega }}\right)>1\Leftrightarrow Deposit,$$ (5)

where $\varphi^{\sigma }=\varphi_{i}^{\sigma }\cdot {\hat{u}}_{i},\;\varphi^{\omega }=\varphi_{i}^{\omega }\cdot {\hat{u}}_{i}$, and we have distinctly labeled the number of neighboring free agents and water nodes as *N*^*σ*^ and *N*^*ω*^, respectively. Scalars *N*^*σ*^*φ*^*σ*^ and *N*^*ω*^*φ*^*ω*^ are numerically measured values that characterize the respective magnitudes of the active and repulsive forces in the direction of motion, and which depend only on the local configuration of the discrete system as illustrated through examples in **Fig 2F–2G**. Therefore, the only parameter introduced through this edge deposition rule is the dimensionless ratio $A=f^{a}/f^{\Gamma }$, which characterizes the competition between a free agent’s self-propulsion force and its effective repulsion from water. As such, A is the parameter that mediates global expansion and shape change of the rafts. Supposing that an ant’s aversion to water is relatively consistent (i.e., that *f*^Γ^ is constant), then A is representative of the active force or “activity” of free ants, where high activity is synonymous with high *f*^*a*^. Increasing A results in an increase in the left-hand side of **Inequality 5**, thereby escalating the overall edge deposition rate per free agent. With the edge deposition rule implemented, a mean expansion (or edge binding) rate of *α*~2% min^−1^ naturally emerged for the overall rafts and automatically matched the experimentally measured values once pseudo-steady state treadmilling occurred (*α*≈*δ*) (**Fig 3D–3F**). This rate may be interpreted as the percentage of structural agents that are newly added to the network’s edge each minute.

### Protrusions emerge spontaneously

To model experiments, we ran simulations with 2,250 agents for up to 4.5 hours of simulation time, letting the rafts reach quasi-steady state treadmilling (defined by *α*≈*δ*). To roughly mimic the dense, spheroidal shapes of the experimental ant aggregations when initially placed into water and to assure that protrusions form stochastically, all simulated rafts were originated as circles with a free agent packing fraction of *ϕ* = 1 freely active agent per structural agent. To provide a still reference frame and mimic the anchored boundary conditions of experimental rafts, a permanent structural agent was located at the center of the domain and fixed in place. With both the pairwise contraction rate ($\dot{d}=1.9\;\%\;{min}^{-1}$) and Vicsek model parameters (*R* = 0.9 ℓ and *η* = 0.2) independently calibrated to match experimental treadmilling and free ant trajectories, respectively, ant activity (A) remained the only free parameter driving freely active agent behavior and global shape evolution. To display ant traffic and transitions between the two layers, **S2** and **S3 Movies** present a simulated raft with structural and freely active agents depicted as light and dark grey particles, respectively, while transition events to and from the structural layer are depicted by cyan and red points, respectively.

**Fig 4:**
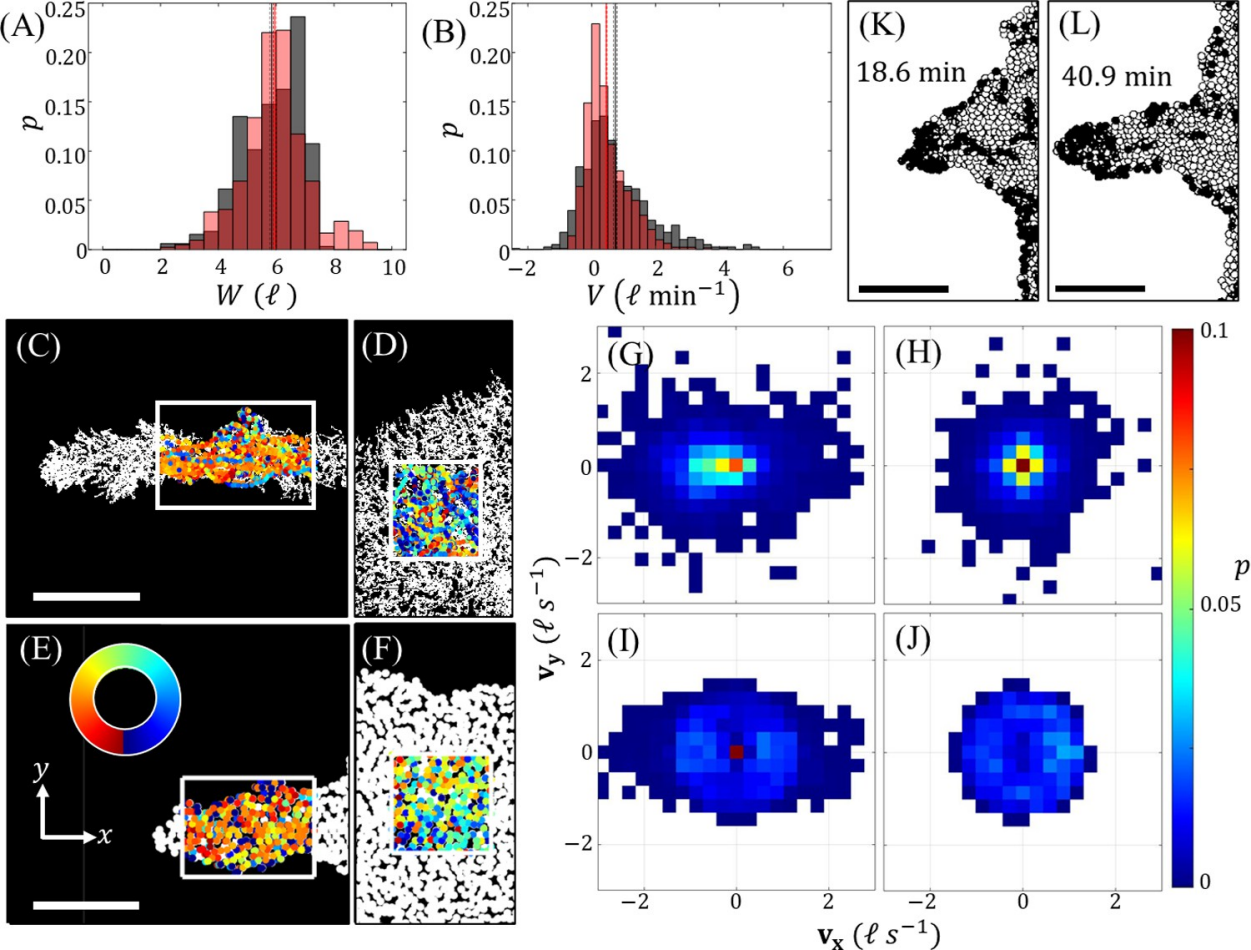
Comparing Protrusion Dynamics. **(A-B)** Probability mass functions are shown for (**A**) the average protrusion widths, *W*, and (**B**) growths rates, *V* of more than 400 experimental observations (grey) and numerical observations (light red) each. Here, *R* = 0.9 ℓ, *η* = 0.2 and A∈[1.25,1.47]. **(C-D)** The direction of motion of free ants on experimental sections of (**C**) a protrusion and (**D**) the bulk of a raft are visually illustrated with the color of a free agent representing its direction of travel during one frame-to-frame observation. **(E-F)** The same visual analysis is made for sections of (**E**) a protrusion and (**F**) the bulk of a simulated raft, where the direction of travel is measured between one timestep. **(C-F)** Colors are assigned according to orientation based on the color wheel depicted in (**E**). (**G-J**) 2D velocity distributions are shown, courtesy of Wagner, et al. (2021) [14]. (**G-H**) correspond to (**C-D**), respectively, while (**I-J**) are the ensembled results from 11 *in silico* protrusions and on the order of 100,000 discrete velocity observations, each. A simulated protrusion at the start **(K)** and end **(L)** of a roughly 21 min duration exhibits how directional motion on protrusions culminates in clustering of freely active agents (black circles) at the tip and rapid, anisotropic growth. **(A-B,C-D,G-H)** Experimental results are courtesy of Wagner, et al. (2021) [14]. Scale bars in (**C,E,K,L**) represent 10 ℓ. All simulated rafts were initiated as circles such that the *in silico* protrusion growths depicted (and from which data were collected) occurred stochastically.

Despite the initially circular shape of each raft, when A was on the order of 1.25 to 1.47 the model consistently predicted the unstable growth of protrusions. For the purposes of this work, we define a protrusion as any elongated region of structural network branching from the raft whose width is less than half of the mean ant persistence length (0.5×*l*_*p*_≈10 ℓ), and whose length is greater than or equal to its width (i.e., aspect ratio ≥1). In contrast, bulk sections of raft are defined as continuous regions of structural network whose dimensions exceed *l*_*p*_ in all directions, and which exists at least a distance of 2 ℓ from the raft’s boundary to account for the correlated trajectory length scale of free ants (~1 ℓ). To determine if the predicted protrusions had the same characteristic length scale and dynamics as experimental protrusions, we measured their average widths, *W*, and growth rates, *V*, over time. The widths of simulated protrusions ranged from roughly 2 to 9 ℓ, with a mean value of 5.95±0.05 ℓ that agrees with the experimental value of 5.85±0.06 ℓ (**Fig 4A**) [14]. Similarly, the tip-growth rates of the model-predicted protrusions ranged from roughly -1 to 3 ℓ min^−1^, with a mean value of 0.46±0.02 ℓ min^−1^ (**Fig 4B**). While not exactly matching the experimental mean of 0.74±0.05 ℓ min^−1^ [14], these growth rates are on the same order and are adjustable through A. The model also allowed us to easily quantify the distinct behaviors of freely active agents, enabling us to confirm the factors leading to spontaneous protrusion initiation and runaway growth.

We confirmed that protrusion initiation is driven by stochastic nucleation of transient ant clusters, which occurred frequently near the rafts’ convex edges, and are primarily attributed to wall-accumulation effects [25,29,40,44–47]. These clusters often caused concentrated edge-deposition of freely active ants resulting in local regions of high edge curvature that served as proto-protrusions. Following this, the model predicted directional flow of freely active ants along protrusions’ lengths consistent with what was observed previously in experiments and which is largely attributed to the strong confinement of particles in these regions wherein their persistence lengths exceed that of the protrusions’ confining widths [14,25,29]. While similar clustering is predicted by the continuum model previously adapted and modified [14] from Fily et al. (2014) [25], several limitations exists for said model, such that the causes of clustering and directional motion in the case of ant rafts are likely better captured by this discrete approach. First, the continuum approach requires a smoothly differentiable raft boundary and predicts that particles will “slide” directly along the edge of confinement until they reach a local minimum in the convex radius of curvature or hit a concave region of edge curvature (at which point they will “jump” tangentially across the domain and back to the opposite boundary) [29]. This smoothing prohibits the study of edge defects whose sizes are on the order of single ants (e.g., vacancies or small protuberances of just a few ants), as the continuum model would predict that these defects simply interrupt sliding along the edge. However, in this coarse-grained lattice model, the raft’s edge defects naturally occur at the length scale of an agent. These defects sometimes interrupted free agent motion along the edge given the movement and edge deposition rules implemented here, causing agents to pause temporarily and then reorient. Despite these defects, the discrete model still predicted directional alignment (**Fig 4C–4F**) and tip clustering (**Fig 4K–4L**) on protrusions, exemplifying robustness in these features, as seen in physical experiments [14]. One possible explanation is that, on protrusions, agents jammed at edge defect sites, were influenced, and frequently realigned with the motion of uninterrupted agents nearby on the bulk such that directional motion resumed. Thus, the effects of volume exclusion, alignment interactions, and bulk movement–none of which could be examined through the continuum approach–propagated the effects of strong confinement inwards (away from the rafts’ edges) and facilitated directional motion.

Visually, directional motion on a protrusion is represented in **Fig 4C–4F** wherein the trajectories of free ants or agents are depicted in regions of interest both on protrusions (**Fig 4C** and **4E**) and bulk sections far from the rafts’ edges (**Fig 4D** and **4F**). The direction of motion is represented by a spectrum of colors per the color wheel in **Fig 4E**, which is oriented such that leftwards movement is depicted by shades of orange. For both experiments and the model, a bias in directional travel is clearly illustrated on protrusions from their bases to their tips, as indicated by the prevailing orange hues of trajectories (here entailing leftwards motion). In contrast, on the bulk it is more difficult to assign a single predominant hue, indicative of more isotropic movement. However, clusters of synchronous motion still appear to occur in all regions of interest on the order of 1 ℓ (consistent with the findings of Wagner, et al. (2021) [14]), making an objective visual analysis difficult. To instead quantify differences in directional motion, we compared the normalized velocity order parameter of free ants and agents (members) on protrusions, defined by *φ* = ⟨***v***(*t*)⟩_*N*_/⟨|***v***(*t*)|⟩_*N*_ where ***v*** is the velocity of a particle and ⟨⟩_*N*_ denotes taking the ensemble average over all *N* members [42]. This parameter is zero when motion is completely isotropic but approaches 1 when movement is perfectly unidirectional. We found that in both experiments and simulations, *φ* was on average higher for freely active members on protrusions than on the bulk of the raft. For the experimental raft depicted in **Fig 4C–4D**, *φ* = 0.65±0.02 on the protrusion versus *φ* = 0.57±0.02 on the bulk [14]. Similarly, for the simulated raft depicted in **Fig 4E–4F**, *φ* = 0.64±0.12 on the protrusion versus *φ* = 0.33±0.06 on the bulk. In both cases, comparably sized domains were used to compute *φ* and the relatively larger values of *φ* on protrusions confirms that the confinement of protrusions induces higher directional motion than that observed on the bulk. However, *φ* does not indicate the orientation or sense of said directional motion.

To further examine in which orientation freely active members preferentially traveled, we also investigated their velocity distributions on and off protrusions, from both experiments (**Fig 4G–4H**) [14] and an ensemble of 11 *in silico* protrusions (**Fig 4I–4J**). The elongation of velocity distributions along the length of protrusions confirms that traffic moves primarily along these structures’ longitudinal axes, whereas the velocity distributions on the bulk (**Fig 4H** and 4**J**) appear uniform, indicating isotropic motion, thus supporting the interpretations of **Fig 4C–4F**. Furthermore, the biased sense of motion is also exemplified by the velocity distributions on protrusions, which are slightly skewed left for both experiments (**Fig 4G**) and simulations (**Fig 4I**), implying motion from the bases-to-tips of protrusions. When directional traffic occurred towards the tips of protrusions, it induced jamming of freely active agents at their ends (**Fig 4K–4L**) and high magnitudes of ***F***^*a*^, similar to the locally high pressures exerted by confined Active Brownian Particles on highly convex regions of confinement curvature [25,48]. This locally high active force accentuated edge binding and tip growth. Ultimately, runaway protrusions result from a positive feedback loop wherein cluster formations initiate protrusions that in turn promote directional traffic, spurring further tip clustering and growth. Indefinite growth of protrusions is checked by both the perpetual raft contraction and finite population of freely active agents, such that within an appropriately large domain the protrusions did not reach the simulation’s boundaries.

### Activity level modulates shape

**Fig 5:**
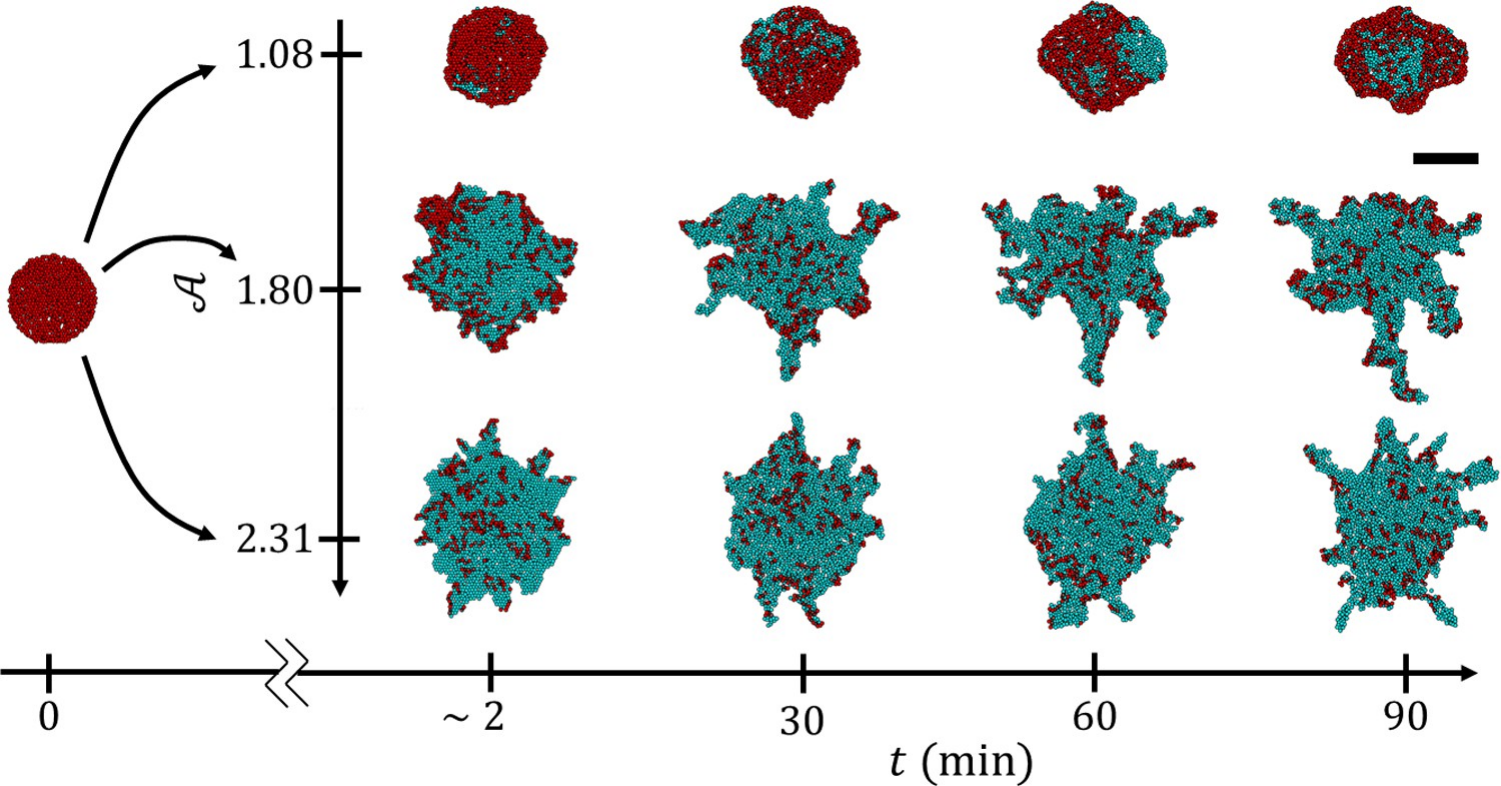
Dynamic Effects of Activity Level. Snapshots of modelled rafts at different simulation times (*t*) and activity levels (A) are depicted to illustrate the effect of A on raft development. The raft on the far left depicts the initial conditions which were the same for each simulation throughout this work (a circular raft with *ϕ* = 1). On the right each row depicts a single raft as it evolves in time (moving from left to right along the horizontal axis). For all simulations shown, *η* = 0.2 and *R* = 0.9 ℓ. Structural agents are depicted in cyan, while dispersed free agents are depicted in red. The scale bar in the top right is universal to all snapshots and represents 14 ℓ.

**Fig 6:**
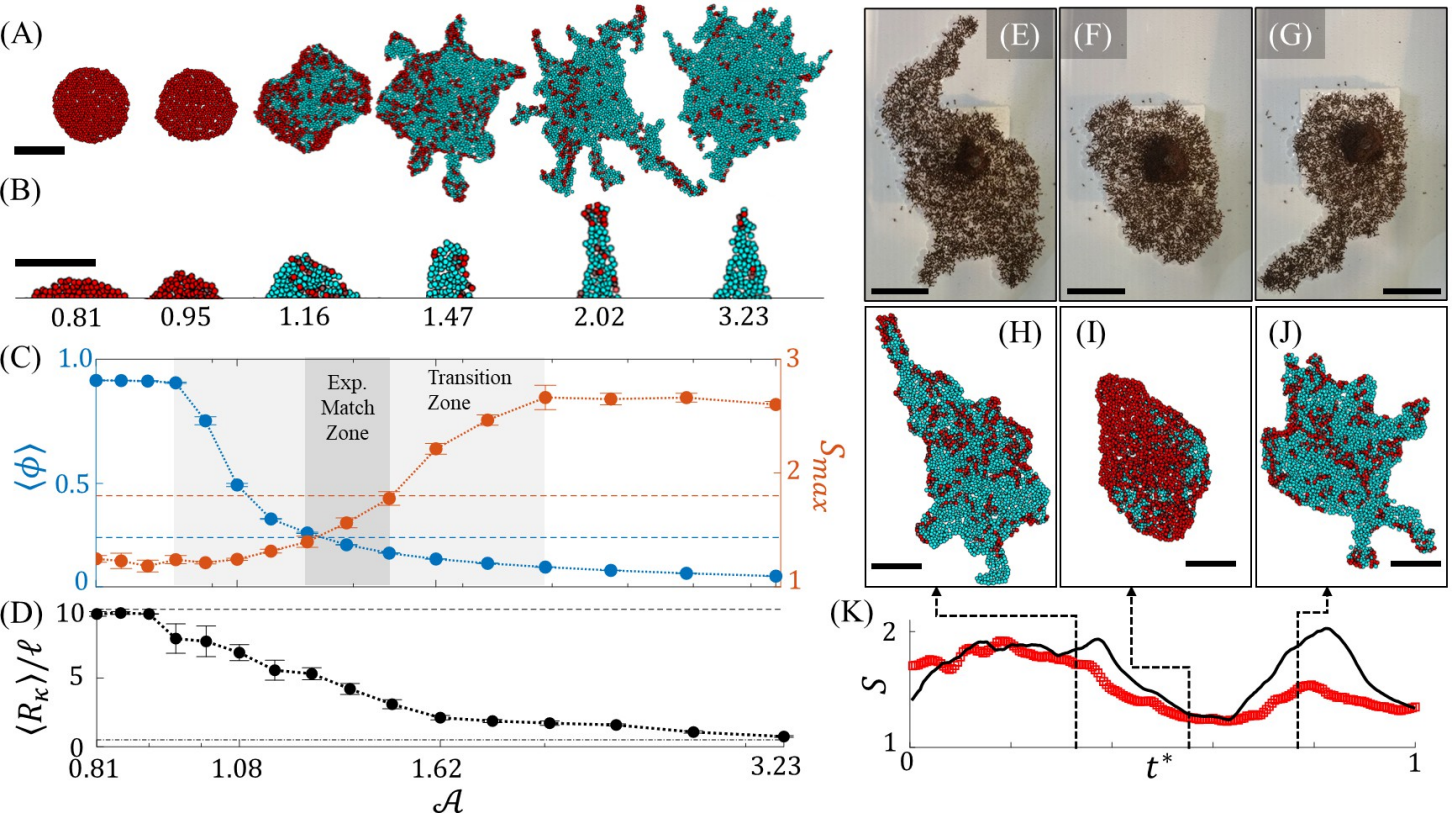
Ant Activity Phases. **(A)** Snapshots of initially circular, simulated rafts are shown after 1.5 hours of simulation time. Here, *η* = 0.2, *R* = 0.9 ℓ and A∈[0.81,3.23]. (**B**) Snapshots of protrusions, each representing the minimum observed radius of tip curvature from its raft at final simulation time, are depicted for each of the respective values of A. The black line cropping each snapshot at its bottom is an open border to the remainder of the raft. The values of A yielding each morphology for (**A-B**) are denoted beneath each snapshot in (**B**). **(C)** Mean freely active agent packing fraction, ⟨*ϕ*⟩, (blue) and maximum surface excess, *S*_*max*_, (red) are plotted with respect to A, and averaged over 5 simulations at each value of A, with error bars presenting standard error of the mean. Horizontal dotted lines in **(C)** represent the experimentally measured values of *ϕ* = 0.24 and *S*_*max*_≈1.8. The bounds of the parameter space that matches experiments are marked where these respective lines intersect the numerical data (see “Exp. Match Zone” between A=1.25 and 1.47). There exists a zone between roughly A=1.0 and 2.0 of continuous phase transition between rafts with minimal-to-no growth whatsoever (*ϕ*≈1 and *S*~1.2) at low activity levels and frequent protrusion growth (low *ϕ* and *S*>2) at high activity levels. **(D)** Mean protrusion tip radius (*R*_*κ*_) is plotted with respect to A. Anywhere from four (in the case of no growth) to forty-one observations were ensemble averaged depending on protrusion frequency. Where no protrusions were available (A≤1.16) the mean convex edge radius is reported instead. The top dotted line represents the initial raft diameter of 10 ℓ, while the bottom dotted line represents the limit of *R*_*κ*_→0.5 ℓ, corresponding to the radius of one agent. **(C-D)** share a horizontal axis. **(E-G)** Three chronological snapshots of an experimental ant raft exhibiting different phases of protrusion growth are compared to (**H-J**) three chronological snapshots of a simulated raft when A was modulated between 1.1 and 1.6. **(K)** The time evolution of surface excess as measured from one experiment (red circles) and ensemble averaged over 28 numerical simulations (black curve with a negligible shaded region representing standard error of the mean) are displayed. Note that the simulations start close to *S* = 1 given the initially circular raft shape. Time, t*, is normalized by the experiment duration for a more direct comparison. Structural agents are depicted in cyan, while dispersed free agents are depicted in red. All scale bars represent 10 ℓ. All simulated rafts displayed were initiated as circles such that protrusions emerged stochastically.

Having confirmed that local-level agent interactions can lead to spontaneous instabilities, we then sought to understand the local behavioral changes that could lead to long-term variation in experimentally observed raft shapes by exploring the effects of A over the range of 0.81 to 3.23 (see **S4–S7 Movies** for samples in this range). Results are summarized in **Figs 5** and **6A** where we visually present the effects of activity on the raft configurations during and after 1.5 hours of simulated time, respectively. From **Fig 5**, we see that no protrusions emerged when A=1.08, whereas protrusions emerged within the first 30 min and 2 min when A=1.80 and 2.31, respectively. Similarly, while there are numerous protrusions stemming from the rafts in **Fig 6A** when A≥1.47, there were no distinct protrusions on the rafts when A≤1.16, based on the definition provided earlier (i.e., width ≤10 ℓ and length ≥ width). This is illustrated in **Fig 6B** by samples of the local edge curvature, each of which displays roughly the smallest geometric edge feature of its respective raft. Generally, these results indicate that higher A promotes higher edge deposition rates that induced more frequent protrusion growth and more eccentric global shapes.

To quantitatively characterize global shape, we introduce a dimensionless parameter called surface excess defined by $S=C/(2\sqrt{A\pi })$, where *C* and *A* are a raft’s perimeter length and area, respectively. *S* = 1 for a circle and increases with a shape’s eccentricity, thus higher *S* indicates the presence of more numerous or more elongated protrusions. Maximum surface excess and mean surface packing fractions of model results are presented in **Fig 6C**. Maximum (as opposed to mean) surface excess is presented to transparently indicate the peak degree of eccentricity achieved by the raft and exclude the inherently low surface excess of the initially circular rafts. Moving from left to right in **Fig 6C** there is a continuous phase transition in the activity range of A=0.95 to 2.02 indicated by the smooth curve of surface excess from low (*S*≈1.2) to high (*S*≈2.8). Likewise, there is a smooth transition of free active agent packing fraction from high (*ϕ*≈1 implying almost no edge binding, whatsoever) to low (*ϕ*≈0.06, indicating relatively high edge binding rates) as A increases. These phases are analogous to those observed in **Fig 1A** and **1B**, respectively.

Besides dictating the global presence of protrusions, A also impacts the local shape of these tethers. Specifically, higher A diminishes the characteristic widths and tip radii of growths as illustrated in **Fig 6B**. To quantify this, the mean protrusion tip radius, ⟨*R*_*κ*_⟩, (or convex edge radius for rafts in which no protrusions emerged) is plotted with respect to A in **Fig 6D**. Examining **Fig 6D**, A≤0.95 distinguishes a region in which ⟨*R*_*κ*_⟩≈10 ℓ, which is approximately the same as the initial raft radius and is exemplary of the lack of growth at low activities. However, for $A>0.95,\;\langle R_{\kappa }\rangle$ decreases monotonically, indicating that regions of lower convex edge radius (or higher curvature, $\kappa \equiv R_{\kappa }^{-1}$) begin to emerge as activity is increased. However, **Fig 6D** confirms that when *A*≤1.16, the edge radii of growths were typically greater than 5 ℓ, suggestive of widths greater than 10 ℓ and mandating that growths such as those depicted in **Fig 6B** for A≤1.16 are not classifiable as protrusions given our prescribed definition. In the region defined by $A>1.16,\;\langle R_{\kappa }\rangle$ appears to approach and eventually reach the limit ⟨*R*_*κ*_⟩→0.5 ℓ, which represents a protrusion tip whose width is just one agent (~1 ℓ) and is therefore the limit in this discrete system.

Mlot, et al. (2011) [12] estimated the capillary length of ant rafts on the order of 10 ℓ. However, from these results we see that raft edge curvature is dependent on activity, and therefore relatable to the length scale, L=l/A. When A is low (*L* is high), we see smoother raft geometries (higher capillary length) with lower surface excess and edge curvature. In contrast, when A is increased, *L* diminishes permitting the emergence of more, but narrower, protrusions. In essence, higher free agent activity reduces effective surface tension of the overall rafts, warranting a comparison of A to temperature [49] in non-active materials whose surface tensions generally diminish as temperature increases [50]. Worth noting is that when A is sufficiently large, the rate and azimuthal homogeneity of edge binding are high enough that expansion appears to approach an isotropic state. This is reflected by the reduction in the number and size of protrusions displayed by the raft in **Fig 6A** when A=3.23. It is likely that as A increases, the propulsion force of a single agent *f*^*a*^ eventually becomes sufficient to cause edge binding anywhere along the raft’s edge, reducing the relative significance of local raft geometry and cooperative force ***F***^***a***^. Significantly, this suggests that there is, in fact, an optimal surface activity level for inducing an exploratory phase in systems that obey this model, which occurs when 1.16<A<3.23.

To demonstrate how A may alter the phases of protrusion growth and non-growth, we modulated A within a given model experiment to a value above (A=1.6) and below (A=1.1) the phase transition threshold (see **Fig 6E–6K** and S8 Movie). Indeed, this effectively toggled the raft between exploratory phases of high surface excess (e.g., **Fig 6H** and **6J**) and low surface excess wherein no protrusions were present (e.g., **Fig 6I**), comparable to what was observed in experiments when free ants ceased activity. Worth noting is that the second phase of experimental protrusion growth (**Fig 6G**) did not reach the same magnitude of surface excess as the initial phase (**Fig 6E**), suggesting that either the activity level did not fully recover to its original state or not enough time was spent in this more active state to resume *S*≈2. Consequently, surface excess of the second simulated phase of protrusion growth (**Fig 6J**), exceeds that of the experimental surface excess. It is reasonable to assume that A evolves roughly continuously for a real ant raft of thousands of individuals, however A in the model was modulated via a binary step function, thus likely contributing to the abrupt resumption of high *S*. Regardless, activity level’s effect on raft shape is made clear.

## Discussion

Our results indicate that fire ant rafts may exhibit spontaneous protrusion growth in the absence of external gradients or long-range interactions. While cueing factors such as pheromones have not been ruled out and should be tested for in future experimental studies, this model generally poses local mechanisms through which fire ants may achieve treadmilling and protrusion growth without centralized control or purposeful intent. Nevertheless, protrusions may sometimes serve the adaptive purpose of helping fire ants escape flooded environments, perhaps illustrating an example in which spontaneous cooperative behavior benefits a collective organism. Through the model, we find that the global shape of these rafts and their display of protrusions is highly dependent on the activity parameter A, which characterizes the competition between an ant’s self-propulsion and its aversion to water. Supposing free ants’ aversion to water does not vary significantly, then A may be interpreted as the normalized force with which free ants self-propel. Inversely, if self-propulsion force is conserved, then increased A may be thought of as a reduced inhibition to structural edge deposition by ants. In either case, the model suggests that a behavioral change by solely the freely active ants may significantly impact the size and shape of ant rafts observed. Tennenbaum and Fernandez-Nieves (2020) demonstrated that temporal activity cycles in fire ants on the order of hours also impact the rheological properties of 3D aggregations. Based on our model, we suspect that this same oscillation of ant behavior between inactive and active states is responsible for the morphological variation in ant rafts. Indeed, in our previous work, we experimentally observed that the clustering of inactive, yet free ants near the centers of their rafts preceded the overall reduction in raft area and reduction in surface excess [14]. During this time, the contraction of the structural layer remained relatively constant.

This model also offers an explanation regarding the directional bias in edge deposition that our previous work indicated is necessary for the evolution of elongated protrusions [14]. We see that strong confinement of active agents on protrusions promotes their directional motion towards the protrusions’ tips, whereas active agent motion is isotropic on the rafts’ bulk sections. Since we enforce that freely active agents deposit into the water in a direction that is correlated with their movement and that of their nearest neighbors (as determined by the local active force, ***F***^*a*^), this directional motion then promotes local edge growth that is aligned with the longitudinal axis of the protrusions. Through this rule, the shapes and growth rates of model-predicted protrusions are in good agreement with those of experiments, thereby supporting the hypothesis that confinement-induced directional motion is a contributing second-order cause of runaway protrusion growth in ant rafts. Ultimately, these results do not nullify the potential influence of biological stimuli (e.g., morphogens or pheromones), rather they support the notion that physics-driven mechanisms may aid or provided a redundant pathway for emergent protrusion growth in ant rafts.

While this discrete model helps interpret potential causes of protrusion growth in ant rafts, there exists several limitations that could influence the accurate representation of free ants by free agent trajectories and therefore global raft evolution. First, free agent movement is restricted to a lattice defined by structural agents and water nodes, which simplifies the model in several ways. For example, it alleviates the need to interpolate a continuous raft boundary, and free agents’ encounters with the water are discretely defined as instances when their preferred movement DOF is a water node. It also renders interpolation of the energy landscape unnecessary at the location of free agents (when computing ***F***^Γ^) since each free agent is already located on a structural agent. Additionally, it permits easy mimicry of volume exclusion between neighboring free agents, by prohibiting two free agents from occupying the same structural site. Finally, it eliminates the need for explicit constrains on agents’ speeds (e.g., frictional forces or inertia) since the agents can move, at most, a distance of *R*_*DOF*_ within a given timestep. However, this lattice naturally introduces a degree of error between the continuously predicted direction of motion from **Eq 1** and the actual direction of discrete movement. This could potentially influence the global raft evolution since it impacts the direction of structural deposition for agents at the raft’s edge. Another discrepancy that could potentially influence the predicted raft evolution is that we opt to treat the ants as particles whose orientational DOF is in-line with their direction of motion. Coupling the translational DOF with the rotational DOF significantly reduces model complexity, yet it effectively surmises that the timescale of alignment is considerably smaller than a discrete timestep. To preserve finite alignment and turning times, we instead restricted the maximum turning angle of an agent within a given step and introduced a pause time in the event that an agent is limited to movements outside this angular range. Additionally, we assume radial symmetry or that that the agents have an aspect ratio of one. In reality, fire ants have an aspect ratio on the order of 3:1 [51,52] and it is well-documented that aspect ratios can introduce alignment effects in self-propelled particles [53]. Indeed, in our previous work [14], we saw evidence of non-negligible ant-to-ant interactions that caused short-range correlation between the motion of nearest neighbors. However, to capture local alignment we opted to employ the relatively simple and phenomenological Vicsek model rather than explicitly modeling an aspect ratio and repulsive interactions. A final limitation that may impact free agent trajectories is that agents were not allowed to walk over one another despite such behavior being regularly observed in freely active ants. This may have exaggerated the effects of volume exclusion between ants and consequently exacerbated any local alignment in velocity by limiting free agents’ local movement degrees of freedom.

Despite these limitations and their potential effects on free agent trajectories, we found that the model sufficiently approximated continuous space when 18 DOF were given to each free agent and the lattice was stochastically updated due to unbinding events and contraction. This is demonstrated by the isotropic distribution of the agent velocities in **Fig 4J** and a general lack in any preferential direction for protrusion growths throughout this work. Furthermore, the mean persistence length of free agents’ trajectories (on the bulk of the modeled rafts), as well as the degree of directional motion (on both bulk sections of the rafts and protrusions) (**Fig 4E–4F**) were both reasonably matched to experiments. However, one ant trajectory feature that remained uncaptured by the model is the frequent jamming of free ants far from the edges of the rafts, as indicated by the peak of the velocity distribution at ***v***≈[0,0] ℓ *s*^−1^ in **Fig 4H**. While free agents in the model were prompted to move at every timestep unless they had no unoccupied DOF, free ants were regularly observed stopping to clean themselves or interact with other ants, regardless of whether their movement was inhibited by obstacles. This discrepancy also appears in the velocity distributions of free ants (**Fig 4G**) versus agents (**Fig 4I**) on protrusions, with simulated free agents generally displaying a much more homogenous distribution of velocities. It is likely that this heightened motility of free agents exaggerates their diffusivity over their ant counterparts; however, the instantaneous distributions of free members–which more directly influence raft evolution–were in good agreement between experiments and simulations.

Another limitation of this model is that it does not currently accommodate inclusion of local cues or external gradients. Such stimuli could enhance the degree of order in the system and if they were introduced by the fire ants themselves (e.g., pheromone trails [30–33,35]; memory over second timescales and centimeter length scales; or collective memory through propagated short range social interactions [54]) they would effectively serve as long-range interaction potentials. Indeed, a precursory study reveals high sensitivity to the pairwise influence length scale *R*, which if set slightly higher (*R* = 1.23 ℓ) leads to the prediction of longer, more ordered protrusion growth at low activities (A=0.81), as depicted in the top right corner of **S6A Fig** (see the **Materials and Methods** section **Extended Parameter Sweep** for details). Moreover, the emergent structural network contraction and unbinding events were simply reproduced here via a homogenized, phenomenological model. Yet the underlying behavioral rules, mechanisms, and the sequence in which they occur are likely far more complex for raft contraction and will be investigated in future work. For both states of ants, there is likely more than one set of rules that results in treadmilling and protrusion growth. Here we have merely investigated a distilled set of local interactions which reproduced the observed raft evolutions, thereby reducing the number of variable considerations and isolating the effects of local ant activity level on global shape. Nevertheless, phenomena such as local pheromones or external temperature gradients, could be easily included in future iterations for the study of not only fire ants but also other constituents. Therefore, although this numerical implementation was inspired by fire ant rafts, we also expect that in future work it may be adapted or inspiration for the *in silico* investigation of other biological or synthetic systems driven by transport and binding reactions. Additionally, this model permits the investigation of emergent phenomena and global characteristics outside the biologically observed parametric space, as occurred here for the purposes of fitting *R* and *η* (see **S5** and **S6 Figs**), and then investigating the effects of A (see **Figs 6** and **S7**). Thus, this model may permit extrapolation of properties and potentially serve as a source of inspiration for the predictive design of engineered systems such as active gels or swarm robotics.

## Materials and methods

Here we provide further details on the numerical implementation of the model, including the algorithmic design and detailed derivations (as necessary), as well as a summary of the model’s parameters, the method by which they were calibrated and extended parameter sweeps of *η*, *R* and A. Finally, methods for computing surface excess and edge curvature are described. All codes and data used to produce this manuscript are deposited in the Dryad repository: https://doi.org/10.5061/dryad.4f4qrfjb3 [55].

### Dryad DOI

https://doi.org/10.5061/dryad.4f4qrfjb3

### General description

#### Domain description

The numerical framework is carried out using MATLAB R2019b. It is a 2D planar, discrete model comprised of distinct nodes defined by some unique index number, *i*∈[1, ∞), and unique Cartesian coordinates, ***X***_*i*_ = [*x*_*i*_, *y*_*i*_]. We locate the nodes inside of a square domain whose center is at position [0,0]. The nodes are initially positioned in a close-packed hexagonal lattice with unit length spacing between nearest neighbors. Each node position is then offset by some random amount in the range [−1/6,1/6] *ζ* in both directions. At initial time, *t* = 0, each node is classified as either a structural agent (shown as cyan circles in **S1 Fig**), or water node (shown as black dots in **S1 Fig**). Structural agents represent structural ants, whereas water nodes represent vacant locations into which free agents may eventually park during edge deposition. To mimic initial experimental conditions, the initial shape of all simulated rafts is a circle with center [0,0] and some prescribed radius (**Fig 5**). Every node within this circular boundary is initially defined as a structural agent. This ensures that any protrusions predicted by the model emerge due to spontaneous symmetry breaking as opposed to through user-enforced asymmetries. Freely active agents are introduced to represent freely active ants (shown as red circles in **S1 Fig**). The initial surface packing fraction is set to 1 free agent per structural agent, although the packing fraction naturally decreases in time as free agents bind into the structural network and a steady state flux of agents to and from each layer is reached. To simulate the movement of free ants on top of the raft, we require that free agents only occupy sites already designated as raft nodes. Additionally, to simplify the model, we enforce volume exclusion between freely active agents so that two free agents cannot occupy the same structural raft site simultaneously.

#### Length scales

Two length scales are referenced in this work. The first is that of the mean ant body length, taken as 1 ℓ = 2.93 mm. Results are generally presented in this length scale for ease of comprehension and comparison to experimental results. However, the numerical model is normalized by a second length scale defined as 1 *ζ* = 1.81 mm. 1 *ζ*^2^ is defined as the area a single ant in the structural raft network occupies (i.e., $1\zeta =\rho_{r}^{-0.5}$, where *ρ*_*r*_ = 0.304 ants mm^−2^ is the planar density of structural ants). This normalization enforces that the density of structural agents is maintained at 1 node per unit area (*ζ*^2^), and the nominal separation between nodes is on the order of 1 unit length (*ζ*).

#### Time scale

We normalize the timescale by taking the average distance a freely active agent travels in one iteration, ⟨*d*⟩, divided by the average experimentally measured free ant speed, *v*_0_ (i.e., Δ*t* = ⟨*d*⟩/*v*_0_).

### Simulating the structural network

The algorithmic chronology used to step the positions of structural agents, implement unbinding events, and update the close-packed positions of water nodes is summarized in **S2 Fig**. In the remainder of this section these processes are described in greater detail.

#### Structural network contraction

Given the experimentally measured contraction rate $\dot{\varepsilon }$, we apply a pairwise strain rate, $\dot{d}$, between connected neighbors within the raft. We do so by updating the pairwise separation vector, ***d***_*ij*_, between all structural agents and their adjacent neighbors residing within some prescribed radius, *R*_*r*_, about the node of interest. We conservatively set to *R*_*r*_ 1.5 ℓ to capture the bridging of raft voids that often occurs between structural ants. *R*_*r*_ = 1.5 ℓ corresponds to roughly 4.5 mm or half the body length of some of the largest fire ants. To implement raft contraction, we update ***d***_*ij*_ at time *t*+Δ*t* according to the forward Euler, exponential decay function:

$$d_{ij}(t+\Delta t)=d_{ij}(t)\exp\left[-\dot{d}\Delta t\right].$$ (6)

Taking ***d***_*ij*_(*t*+Δ*t*) as the targeted equilibrium separation at time, *t*+Δ*t*, we then employ an overdamped approach to iteratively step the position of the nodes. The updated pairwise separation vector between neighbors *i* and *j* at iteration *k* is denoted by $d_{ij}^{k}=X_{i}^{k}-X_{j}^{k}$, where $d_{ij}^{1}=d_{ij}(t)$ represents the initial separation at the start of the timestep. The iterative change in position is then given by:

$$\Delta d_{ij}^{k+1}=d_{ij}^{k}-d_{ij}(t+\Delta t).$$ (7)

The position of each structural agent is then updated according to:

$$X_{i}^{k+1}=X_{i}^{k}+\nu^{-1}\sum_{j}\Delta d_{ij}^{k+1},$$ (8)

where *ν*∈[1, ∞) is simply a pseudo-viscosity or over-damping scalar used for computational stability and $\sum_{j}\Delta d_{ij}^{k+1}$ is the net displacement due to all pairwise neighbors. The over-damping scalar was set such that residual displacements through $\sum_{j}\Delta d_{ij}^{k+1}$ converged towards zero. Given the updated coordinates $X_{i}^{k}$, we then re-calculate $d_{ij}^{k}$ for each neighbor, and iterate **Eq 7** and **8** until the residuals dip below some prescribed threshold. Here we define the residuals and their thresholds as $\max\left[\Delta d_{ij}^{k+1}\right]\leq 5\times 10^{-5}\;\zeta$ and $mean[\Delta d_{ij}^{k+1}]\leq 1\times 10^{-5}\;\zeta$.

#### Close-packing water nodes

Since the rafts’ structural networks contract in time, we need to ensure that water nodes remain closely packed to their perimeters so that binding events of freely active agents remain possible at the edge. To do this, we apply a radial linear velocity gradient to all water nodes that moves them towards the center of the domain, [0,0], at the rate of $\dot{d}$, according to $\Delta d_{i}(t+\Delta t)=d_{i}(t)\{1-\exp\left[-\dot{d}\Delta t\right]\}$, where ***d***_*i*_ is the separation vector of each water node with respect to [0,0]. To evenly space water nodes from each other, as well as the rafts’ edges, we introduce Gaussian, pair-wise repulsive forces, $F_{ij}^{r}$, between water nodes and their nearest structural agent or water node neighbors of the form:

$$F_{ij}^{r}=\kappa \frac{{\hat{d}}_{ij}}{\sigma \sqrt{2\pi }}\exp\left[-\frac{{\left(d_{ij}-\mu \right)}^{2}}{2\sigma^{2}}\right],$$ (9)

where *σ* is the standard deviation of the curve, *μ* = 0 *ζ* is the mean, and *κ* is a scaling factor in units of pseudo-force. We use these values to step the positions of water nodes according to:

$$X_{i}(t+\Delta t)=X_{i}(t)+\frac{\Delta t}{\nu }\sum_{j}F_{ij}^{r},$$ (10)

where *ν*∈[1, ∞) is simply another pseudo-viscosity for computational stability, and $\sum_{j}F_{ij}^{r}$ is the net repulsive force due to all pairwise neighbors. Again, the pseudo-viscosity was set such that residual forces through $\sum_{j}F_{ij}^{r}$ converged towards zero. We found that *σ* = 0.5 *ζ* and *κ*/*ν* = 0.02 provided a stable computational domain in which water nodes remained close-packed in an evenly distributed point field (as displayed in **S1 Fig**), thus offering ample water DOF, for freely active agents on the edge of the raft to deposit into. Note that the repulsive interactions between water nodes and their raft neighbors were one-way such that the structural agents could displace water nodes, but water nodes could not displace structural agents. This was done because the close packing of water nodes is a numerical method implemented to homogenize the domain (rather than any physical phenomena) and should not influence the position of structural agents in the model.

#### Unbinding to maintain structural density

Recall that we normalize the domain’s unit length by *ζ* such that the domain’s nominal density, *ρ*_*d*_, is approximately 1 node *ζ*^−2^. Since we observed that structural network density remains roughly constant, we enforce unbinding events in simulations when the domain density exceeds 1 node *ζ*^−2^. To ensure that nodes are removed from the densest locations with precision, we subdivide the domain into a square grid whose unit cell lengths, *L*_*g*_, are ≥1*ζ*. The number of permissible nodes, *N*_*p*_, within each grid cell becomes $N_{p}=\rho_{d}L_{g}^{2}$. For our purposes, we found that *L*_*g*_ = 2*ζ* and *N*_*p*_ = 4 nodes provided sufficient regional discretion to maintain a homogeneous domain (as seen in **S1 Fig**).

After contraction but prior to the stepping of free agents, we conduct a count of the number of nodes occupying each grid site and if it exceeds *N*_*p*_, we initiate a node deletion event. To introduce further specificity in which nodes to remove we calculate the pair-wise distance, *d*_*ij*_, between each node in the pertinent grid space. If both nodes *i* and *j* belonging to the smallest value of *d*_*ij*_ occupy the grid space, one of the two is randomly selected for deletion. In the case that the removed node is a water node, we simply delete it. Stochastic deletion is counteracted by the enforced close-packing described in the previous section and together these practices ensure that the density of water nodes is equivalent to that of the structural raft. New water nodes are seeded at the edge of the domain as needed to conserve their population. However, if the removed node is occupied by a structural agent, we convert it to a free agent positioned at the coordinates of the nearest empty structural node. This introduces unbinding of structural agents into the freely active layer wherever the local network density is high, consistent with what was observed experimentally [14]. By counting the number of unbinding events, *N*_*u*_, at each time step and normalizing by the total number of structural ants, *N*_*r*_, we can calculate the unbinding rate according to *δ* = *N*_*u*_/(*N*_*r*_Δ*t*).

### Simulating the freely active ants

The algorithmic chronology used for determining movement of freely active agent *i* is summarized in **S3 Fig**. As illustrated in **S3 Fig**, the movement of free agents is updated after the contraction and unbinding outlined in **S2 Fig**. In the remainder of this subsection, we detail the rules by which free agent motion is stepped, and then derive **Eq 5** for the effective pairwise edge repulsion felt by an agent due to a water node.

#### Stepping free agent movement

Mirroring the methods of Couzin and Franks (2003) [35] or Baumgartner and Ryan (2020) [34], we assign each free agent a preferred angle of movement, *θ*_*i*_, (as measured from the positive horizontal axis) prior to stepping its position. Here, this is achieved through the Vicsek model (**Eq 1**) to capture experimentally observed local alignment effects [14]. Note that the directions of motion of free agents are dependent on those of the previous time step. Therefore, to initiate the movement of free agents at the start of the simulation (or whenever a structural agent transitions to a free agent) we assign each agent a random, instantaneous orientation, *θ*_*i*_∈[0,2*π*] radians.

With the preferred angle of motion predicted, we assign every agent in the domain 18 DOF, which–in an equidistant hexagonal lattice–corresponds to two layers of nearest neighbors (here spanning 2 *ζ*). We opted to provide each node with 18 DOF based on three considerations: (1) the experimental observation that free ants frequently walk over one another, effectively passing 2 *ζ* in one unit time; (2) the experimental observation that freely active ants may walk over voids in the raft of comparable dimensions to their own body length, effectively passing greater than 1 *ζ* in one unit time; and (3) the realization that modeling free agents with 18 rotational DOF is roughly a threefold improvement in approximating the continuous space real ants occupy, over the 6 DOF offered by looking at only one layer of immediate node neighbors spanning 1 *ζ*. The 18 DOF for each node are assigned in rank-order by distance to neighboring nodes. To be a DOF, the neighboring node must reside within the distance, *R*_*DOF*_∈(0,2.5] *ζ*, of the node of interest. To consistently achieve 18 DOF despite noise in the node distribution, the upper bound of this range was set 1.25 × greater than the distance needed to reach 18 nearest neighbors in a close-packed hexagonal lattice.

With the pool of DOF defined, we then calculate the relative angle, *ϑ*_*ij*_, of each *j*^*th*^ DOF with respect to the position of node *i* as measured with respect to the positive horizontal axis according to:

$$\vartheta_{ij}=\left\{ \begin{array}{ll} atan2(X_{j}-X_{i}), & y_{j}\geq y_{i}\\ 2\pi +atan2(X_{j}-X_{i}), & y_{j}<y_{i} \end{array}\right.,$$ (11)

where ***X***_*j*_ is the position of each DOF, ***X***_*i*_ is position of the freely active agent, and *y*_*i*_ denotes the y-axis component of ***X***_*i*_. We then calculate the absolute difference between *θ*_*i*_ and *ϑ*_*j*_:

$${\Delta \theta }_{ij}=\left\{ \begin{array}{ll} |\vartheta_{ij}-\theta_{i}|, & |\vartheta_{jj}-\theta_{i}|<\pi \\ 2\pi -|\vartheta_{ij}-\theta_{i}|, & |\vartheta_{ij}-\theta_{i}|\geq \pi \end{array}\right.,$$ (12)

and take the minimum value to indicate which neighboring raft or water node the freely active agent would preferentially move to. The pool of DOF are then rank-ordered from smallest to largest Δ*θ*_*ij*_ and sequentially checked for eligibility of movement. All eligible DOF must reside within the turning limit *ϑ*_*j*_∈[−*π*/2, *π*/2] as discussed earlier. Structural DOF are only eligible if they are unoccupied by other free agents. Water DOF are eligible only if the edge deposition condition (**Inequality 5**) is satisfied. The free agent is stepped to the first DOF in the rank-ordered pool that proves eligible. In the case that no DOF are eligible, the agent pauses according to the following section. To eliminate bias and ensure randomization, the order in which the free agents’ motions are updated is randomly determined at every timestep.

#### Pausing surface traffic

While updating the positions of a freely active agent, we run into cases where the pool of potential movement DOF is exhausted, and movement is interrupted. This occurs when all the DOF within the turning limit (*ϑ*_*ij*_∈[−*π*/2, *π*/2]) are either occupied structural agents, or water nodes that do not satisfy the edge deposition condition (**Inequality 5**). We generally observe that real ants whose trajectories are interrupted tend to pause and stationarily explore the environment in front of them for approximately 1 to 10 s before turning around (i.e., turning greater than *π*/2 radians) to explore elsewhere. Therefore, in cases where no DOF meet the set of eligibility criteria and the free agent’s motion is interrupted, the agent is paused for some random time, *t*_*p*_, in the range of [2,4]Δ*t*. After this time has elapsed, the free agent’s orientation is randomly redefined according to the process used at particle initiation and the agent is permitted to resume motion in an uncorrelated direction. This mechanism generates traffic jams in confined regions, such as areas with higher local free agent densities and those confined by the rafts’ edges (e.g., the tips of protrusions). Yet this also ensures that these traffic jams (or clusters) are not permanent features in the simulations. Instead, they dissipate at time scales correlating to both *t*_*p*_ and the cluster or traffic jam size, thus ensuring that clusters occur as they do in experiments.

#### Effective repulsion at the edge

A freely active agent is defined as encountering the edge of the raft when its preferred DOF is a water node. This is tantamount to it encountering the water head-on, and amounts to an initial perception cone [31,35] of roughly ±15° given the 18 movement DOF in this framework. However, once a water node is detected as the preferred movement DOF, the full 360° environment within detection radius *R* is considered. This treatment serves to capture two experimentally observed tendencies of free ants: (1) that free ants moving tangent to but directly near the raft’s perimeter seem unperturbed by the water’s presence to their left or right, and (2) free ants whose movement is halted by encountering water head-on tend to reach out and probe their environment each direction before making a movement decision. In any case, the local change in substrate at the edge of the raft results in some effective edge repulsion for those free agents which detect it. To model this edge force, we define some energetic potential at the site of every structural agent (*U*_*ρ*_), and a another at the site of every water node (*U*_*ω*_). These potentials will map the energy landscape whose local gradient (−∇_***r***_*U*) represents the edge repulsion force ***F***^Γ^.

To gauge this landscape, we first consider the 1D pairwise energy gradient between a freely active agent at node *i* and its *j*^*th*^ detected neighbor (reference **S4 Fig**). Applying the simplest assumption that the gradient between each pairwise set of nodes evolves linearly, then the magnitude of the local pairwise force occurring on a free agent at node *i* because of node *j* is given as *f*_*ij*_ = −Δ*U*_*ij*_/*r*_*ij*_ where Δ*U*_*ij*_ is the step in energy from nodes *i* to *j* and *r*_*ij*_ is the distance between their centers. We make the distinction between structural and water neighbors by replacing the index *j* with *ρ* and *ω* to represent structural and water nodes, respectively, such that two separate types of forces emerge: forces due to adjacent water nodes, *f*_*iω*_ = −Δ*U*_*iω*_/*r*_*iω*_, and forces due to adjacent structural nodes, *f*_*iρ*_ = −Δ*U*_*iρ*_/*r*_*iρ*_. Note that a freely active agent always occupies a structural site, such that the local energy is *U*_*ρ*_. Therefore, Δ*U*_*iω*_ = *U*_*ω*_−*U*_*ρ*_, which we denote as Γ throughout this work (**S4A Fig**). This also means that Δ*U*_*iρ*_ = *U*_*ρ*_−*U*_*ρ*_ = 0 and no effective force emerges due to neighboring structural sites (**S4B Fig**). In 2D, we consider that a freely active agent detects all neighboring nodes within detection distance *R* so that the effective edge repulsion experienced is approximately equal to the sum of all pairwise contributions from nearby water nodes, or:

$$F_{i}^{\Gamma }=-\nabla_{r}U\approx -\Gamma \sum_{\omega }r_{i\omega }^{-1}{\hat{r}}_{i\omega },$$ (13)

where ${\hat{r}}_{i\omega }$ is the direction of the pairwise separation vector ***r***_*iω*_. In the numerical framework, wherein *R* is on the order of the contact length scale (i.e., *R*/ℓ~1, as determined experimentally [14]) we recognize that the separation distances, are all given by *r*_*iω*_≈*R*. Thus, we may take Γ/*r*_*iω*_ as a constant, *f*^Γ^ = Γ/*R* and rewrite **Eq 13** as:

$$F_{i}^{\Gamma }=-f^{\Gamma }\sum_{\omega }{\hat{r}}_{i\omega }.$$ (14)

### Model parameters

A summary of experimental and numerical values used throughout this work is provided in **Table 1**. Experimental values of **Table 1** were taken from Wagner, et al. (2021) [14].

**Table 1:** Commonly referenced values.

Symbol	Definition	Value	Units	Purpose
*ρ*_*r*_	Planar density of structural ants.	0.30	ants mm^-1^	Used to define normalized length scale of model *ζ*.
*ζ*	Occupancy length scale of one structural ant, $\zeta =\rho_{r}^{-0.5}$.	1.8	mm	Defines model length scale.
ℓ	Average body length of one ant	2.9	mm	Used to normalize length scale of results.
$\dot{\varepsilon }$	Contractile strain rate of the structural ant network.	1.6	% min^-1^	Used to calibrate the pairwise contraction rate, $\dot{d}$.
*δ*	Exit rate of structural ants into the freely active layer.	~2−3	% min^-1^	Matched between model and experiments to validate choice of $\dot{\varepsilon }$ and exit threshold density *ρ*_*thresh*_.
*l*_*p*_	Walking persistence length of free ants.	~20	ℓ	Used to calibrate noise parameter, *η*.
*v*_0_	Mean free ant speed.	0.97	*ζ* s^-1^	Used to define timescale of model, Δ*t*.
⟨*d*⟩	Average distance traveled by free agent in one time step.	1.67	*ζ*	Estimated as mean distance between adjacent movement DOF. Used to define Δ*t*.
Δ*t*	Discrete time step size, Δ*t* = ⟨*d*⟩/*v*_0_.	0.6	s	Defines model time scale.
*α*	Deposition rate of free ants into the structural layer at the edge.	~2−3	% min^-1^	Used to identify when simulated rafts reached steady state treadmilling (when *α*≈*δ*).

A summary of the model’s free parameters is provided in **Table 2**. The structural agent model contains just one free parameter, the contraction rate between nearest neighbors ($\dot{d}$). However, this value was fixed to reproduce the global contraction rate, $\dot{\varepsilon }$, of experimentally observed rafts [14]. The freely active agent model has three free parameters: (1) the radius of mutual influence between an agent and its nearest neighbors (*R*); (2) activity (A); and (3) the noise parameter (*η*). However, *R* and *η* were fixed to match the walking characteristics of experimental ants (See the **Model Calibration** section, below).

**Table 2:** Free parameters of model.

Phase	Parameter	Type	Definition	Value	Units	Calibration
Structural State	$\dot{d}$	Fixed	Pairwise contraction rate between structural agents.	1.9	% min^-1^	Fixed to reproduce experimental global contraction rate.
Freely Active State	*R*	Fixed	Radius of mutual influence of surface agents.	≤1	ℓ	Experimentally estimated as contact length scale between neighboring ants.
	*η*	Fixed	Noise parameter ∈[0,1]	0.2	N/A	Fixed to reproduce experimental free ant trajectory persistence length.
	*L* (of A)	Swept	Length scale defined by *L* = Γ/*f*^*a*^. Controls activity parameter through A=l/L.	*L*∈[0.5,2], A∈[3.2,0.8]	ℓ	Swept

### Model calibration

#### Pairwise strain

The numerical contractile strain rate was controlled using the parameter, $\dot{d}$, employed according to **Eq 5**. We calibrated $\dot{d}$ by matching the global contractile strain and numerical exit rates ($\dot{\varepsilon }$ and *δ*, respectively) to those of the experiments. Global decay was measured experimentally using particle image velocimetry on a rectilinear region of interest over a duration of 13 minutes and calculating the radial component of contractile speed, $\dot{r}$, towards the still reference frame (i.e., the stationary acrylic rod). A linear gradient in $\dot{r}$ was found with respect to distance away from the stationary acrylic rod, *r*, suggesting a spatially constant $\dot{\varepsilon }$ [14] (**Fig 3A and 3C**). For a given experiment, $\dot{\varepsilon }$ was also found to be roughly constant in time, and isotropic [14]. The combination of spatially constant $\dot{\varepsilon }$ and isotropic contraction indicates that the mechanism of contraction occurs locally and homogenously throughout the bulk of the raft, rather than at a specific location such as the interface between the raft and the rod [14], hence the use of a locally applied pairwise contractile strain rate between neighboring structural agents. For the numerical results, $\dot{\varepsilon }$ was also computed as the gradient in contractile speed towards the stationary raft point with respect to distance from said point (**Fig 3B and 3C**). We found that $\dot{\varepsilon }$ matched between the model and experiments when $\dot{d}$ was set to 1.2 × the desired global strain rate, likely due to affine effects within the network structure [39]. Note that the results presented in **Fig 3C** of both experimental and simulated raft contraction represent the full data of 2D regions of interest but are projected onto one dimension (which is radial distance from the still reference point, *r*).

### Vicsek model parameters

The effects of altering *R* and *η* are illustrated through the phase table in **S5 Fig**, wherein the surface traffic of freely active agents’ in 2D domains with periodic boundary conditions is depicted at various values of these parameters. Note that to correctly mimic the conditions of the lattice model used in the raft framework, the agents are here also restricted to motion on a lattice, whose positions are set in a hexagonal close-packed configuration and then randomly offset by some amount ∈[−1/6,1/6] *ζ* in each direction, as described above in the section titled, **Domain Description**. Agent motion is also restricted to 18 DOF at every timestep and governed by the rules described in the section titled **Simulating the Freely Active Ants**. Moving from left to right, we see that the effect of decreasing *η* (or decreasing the rotational noise, *ξ*_*i*_ in **Eq 1**) is to induce collective motion and directional flow. Likewise, moving from top to bottom, we see that increasing *R* (or increasing the range over which freely active agents are influenced by their neighbors) has a similar effect.

From our previous work [14], we are aware that *R* is on the order of 1 ℓ, which gives us an estimation of the initial length-scale for the numerical value. Additionally, we found that the free ant trajectory persistence length, *l*_*p*_, on the bulk of the raft is roughly 15−20 ℓ. Employing the method used in Wagner, et al. (2021) [14], we also calculate *l*_*p*_ for of simulated freely active agents in the parameter space given from **S5 Fig**, yielding the heat map illustrated in **S6 Fig**. Matching *R* and *l*_*p*_ to the approximate experimental values of 0.9 ℓ and 15 ℓ, respectively, we find that *η*≈0.2.

### Extended parameter sweep

With *η* set to 0.2, A was swept over the range [0.81,3.24]. The results presented in this manuscript were produced by fixing *R* at 0.9 ℓ to mimic experimental systems and examine the effects of local interactions only. However, *R* was supplementarily swept over the range of [0.62,1.23] ℓ to elucidate its effects on overall raft shape. Note that *R*∈[0.62,1.23] ℓ corresponds to *R*∈[0.5,2] *ζ* and A∈[3.24,0.81] corresponds to *L*∈[2,05] *ζ*, hence the respective choices for *R* and A in this work. The extended phase table and heat maps of free agent packing fraction, *ϕ*, and peak surface excess, *S*_*max*_, are depicted in **S7A**, **S7C** and **S7D Figs**, respectively. **S7B Fig** depicts the combinations of *R* and A that result in matching of *S*_*max*_ (red curve) and *ϕ* (black curve) to those values from the experiments. While the two curves never intersect in the parameter space, this may be attributed to several factors including error in the numerical measurement of surface excess and isotropic detection of neighboring agents within detection radius *R*.

### Measuring surface excess

Recall that surface excess is calculated according to the relation $S=C/(2\sqrt{\pi A})$, where *C* is the raft’s perimeter length and *A* is the raft’s area. The way in which *C* is measured may significantly impact the estimated value of *S* due to the fractal nature of ant rafts’ edges. Specifically, if the contour length is measured with resolution better than the length scale of the constituents’ size, then *C* captures the surface roughness of the raft edge and is overestimated in accordance with the coastline paradox [56]. As such, a manual method of measuring *C* by tracing the perimeter of the raft in each frame using ImageJ [57–59] was preferred, as it allowed user discretion in capturing edge defects. This method was used for the experimental dotted line presented in **Fig 6C**, which coarsely estimates the maximum experimental surface excess on the order of 1.8.

Surface excess of numerical results was estimated by taking *C* as the number of structural agents on the perimeter and *A* as the total number of structural agents. This estimation of *A* is acceptable since the domain density is maintained at 1 node *ζ*^−2^, meaning each structural agent occupies a space of 1 square unit length. Similarly, this estimation of *C* relies on the fact that the nominal spacing between nodes is ~1 *ζ*, such that adjacent structural agents in the perimeter may be assumed approximately 1 unit length apart. Perimeter structural agents were defined as agents with neighboring water nodes that reside within the threshold distance of nearest neighbors (i.e., are ≤1 ℓ or ≤1.6 *ζ* away). This method was used for expediency as it could be automated during simulation post-processing for the ensemble average presented in **Fig 6C**. However, the assumption of unit spacing (1 *ζ*) between adjacent edge agents may introduce error in the calculation of *C*.

To directly compare surface excess between experimental and model results, as done in **Fig 6K**, an alternative and controlled method was used. Both experimental and numerical videos of the raft evolution were imported into ImageJ and converted to binary images with the raft black and the background white. All black pixels that were not part of the raft were removed such that the raft was the only object in the image. Each image was then eroded twelve times to reduce surface roughness at the length scale of individual ants or agents, and then dilated twelve times to revert the rafts back to their original size. The image was then analyzed to measure *A* and *C* and calculate surface excess. Using this method for both the experimental and numerical results permits a more direct comparison between the two image sources.

It should be noted that regardless of the method employed, the fractal nature of ant rafts’ “coasts” ensures that surface excess is strongly impacted by the resolution with which *C* is measured [14]. While surface excess quantifies shape to some extent, it is here used to interpret qualitative and relative changes in global raft shape rather than draw absolute or quantitative conclusions.

### Measuring Tip Radii

To measure the radii of curvature of *in silico* rafts’ convex edges, the final frames of simulated rafts (for A∈[0.81,3.23]) were uploaded into ImageJ [57–59]. Convex regions of edge curvature were visually identified, and the positions of each structural agent in the local vicinity were tracked using ImageJ’s “point tool”. Convex regions were mathematically delineated from their adjacent concave regions by inflection points in the local curvature with a moving average interval of (1 ℓ). Data outside the nearest inflection points was cropped. A circle was then fit to the remaining convex point data using the Pratt method [60,61]. The radius of this circle was taken as the local radius of curvature, *R*_*κ*_. If only one or two points remained within the dataset after cropping between inflection points (in which case the Pratt method would not work), then these regions were confirmed to contain just one or two agents and the corresponding radius was set to 0.5 ℓ or 1 ℓ, respectively. Once computed for every observation, the mean of all observations (⟨*R*_*κ*_⟩) and standard error of the mean were calculated and reported in **Fig 6D**.

## Supporting information

S1 Fig Domain Depiction. A snapshot of the discrete numerical domain is shown with water nodes plotted as black dots, condensed structural agents plotted as cyan circles, and dispersed freely active agents plotted as red circles.
(TIF)

S2 Fig Algorithmic Chronology of Structural Agents and Water Nodes. A flow chart details the algorithmic order in which positions of structural agents and water nodes are updated. The point at which unbinding events occur is also displayed.
(TIF)

S3 Fig Algorithmic Chronology of Freely Active Agents. A flow chart details the algorithm by which movement is determined for each freely active agent, in each time step.
(TIF)

S4 Fig Pairwise Repulsive Force. **(A)** The origins of the pairwise repulsive force acting at the position of structural node *i* due to the proximity of water node *ω* is illustrated in 1D. The force is taken as the gradient in energy landscape from *ω* to *i*. **(B)** Similarly, the lack of any pairwise repulsive force acting at the positions between structural node *i* and structural node *ρ* is visually illustrated by the lack of a gradient in the local energy.
(TIF)

S5 Fig Vicsek Model Phase Diagrams. Snapshots of the surface traffic of freely active agents in the numerical model are illustrated at a packing fraction of *ϕ* = 0.24. The **(A)** full traffic, as well as **(B)** streamlines of just 10% of modeled agents composited from 10 time steps, are shown to illustrate the presence of clustering and directional motion, respectively. From top to bottom *R* is swept over the range *R*∈[0.62,2.47] ℓ and from left to right, *η* is swept over the range *η* = [1.00,0] in increments of 0.25. The regional range that roughly matches experiments is outlined in red for each table. Although the agents’ motions are confined to a lattice of nodes, the lattice is not depicted here for visual clarity.
(TIF)

S6 Fig Persistence Length Phase Diagram. An interpolated, 2D heat map illustrates how *l*_*p*_ evolves over the parameter space defined by *R*∈[0.62,2.47] ℓ and *η*∈[0,1] in the numerical model. The point that matches the experimental data is plotted as a white dot.
(TIF)

S7 FigExtended Parameter Sweeps and Phase Diagrams.**(A)** A phase table depicts the morphology of simulated rafts for different values of *R* and A after approximately 1 hour of simulated time. **(B)** Interpolated curves with respect to *R* and A depict the phase space in which the maximum surface excess *S*_*max*_ (red) and packing fraction *ϕ* (black) matched those of the experiments (~1.8 and ~0.24, respectively) to within 0.25%. **(C)** A heat map of *ϕ* with respect to *R* and A is shown with the white curve corresponding to the black curve from **(B)**. **(D)** A heat map of *S*_*max*_ is shown with respect to *R* and A with the white curve corresponding to the red curve from **(B)**.(TIF)

S1 Movie **Experimental Treadmilling** presents raw, time-lapsed video footage of an experimental ant raft to visually demonstrate the treadmilling and protrusion growth that takes place. The scale bar represents 10 ℓ and a timestamp is included in the top right.
(MP4)

S2 Movie **State Transitions** depicts a simulated ant raft comprised of 2,250 agents over a span of 175 simulation minutes at approximately 360x speed to clearly illustrate the transition events between the free active layer and structural layer. Within each frame, transitions from the freely active layer to structural layer are denoted by cyan circles, while transitions from the structural layer to the freely active layer are denoted by red circles. Non-transitioning structural agents are denoted by light and dark grey particles, respectively. Freely active agents are not depicted to reduce noise and improve visual clarity. The raft was initiated as a circle.
(MP4)

S3 Movie **Surface Traffic** displays a snippet of **S2 Movie** to that spans 70 simulation minutes at approximately 60x speed in order to clearly display the movement of freely active agents. As with **S2 Movie**, transitions from the freely active layer to structural layer are denoted by cyan circles, and transitions from the structural layer to the freely active layer are denoted by red circles. Non-transitioning structural and freely active agents are denoted by light and dark grey particles, respectively.
(MP4)

S4 MovieIllustrates simulated ant rafts comprised of up to 2,250 agents over a span of 4.5 simulation hours. All rafts were initiated as circles and simulated until pseudo-steady state treadmilling occurred (wherein *α*≈*δ*). In all simulations, *R* = 0.9 ℓ and *η* = 0.2. A was set to 1.08, 1.42, 1.80, and 3.24 for S4–S7 **Movies**, respectively. Note that a finer separation in A between videos is provided in the range 1.35≤A≤1.59 since this is the approximate range of continuous phase change identified. All scale bars represent 40 mm or ~14 ℓ. The rafts were initiated as circles. Note that these videos are displayed over long durations at approximately 1500x speed. Therefore, the individual transition of agents from one state to another, as well as the motion of freely active agents (red) are not immediately distinguishable.(MP4)

S5 MovieIllustrates simulated ant rafts comprised of up to 2,250 agents over a span of 4.5 simulation hours. All rafts were initiated as circles and simulated until pseudo-steady state treadmilling occurred (wherein *α*≈*δ*). In all simulations, *R* = 0.9 ℓ and *η* = 0.2. A was set to 1.08, 1.42, 1.80, and 3.24 for S4–S7 **Movies**, respectively. Note that a finer separation in A between videos is provided in the range 1.35≤A≤1.59 since this is the approximate range of continuous phase change identified. All scale bars represent 40 mm or ~14 ℓ. The rafts were initiated as circles. Note that these videos are displayed over long durations at approximately 1500x speed. Therefore, the individual transition of agents from one state to another, as well as the motion of freely active agents (red) are not immediately distinguishable.(MP4)

S6 MovieIllustrates simulated ant rafts comprised of up to 2,250 agents over a span of 4.5 simulation hours. All rafts were initiated as circles and simulated until pseudo-steady state treadmilling occurred (wherein *α*≈*δ*). In all simulations, *R* = 0.9 ℓ and *η* = 0.2. A was set to 1.08, 1.42, 1.80, and 3.24 for S4–S7 **Movies**, respectively. Note that a finer separation in A between videos is provided in the range 1.35≤A≤1.59 since this is the approximate range of continuous phase change identified. All scale bars represent 40 mm or ~14 ℓ. The rafts were initiated as circles. Note that these videos are displayed over long durations at approximately 1500x speed. Therefore, the individual transition of agents from one state to another, as well as the motion of freely active agents (red) are not immediately distinguishable.(MP4)

S7 MovieIllustrates simulated ant rafts comprised of up to 2,250 agents over a span of 4.5 simulation hours. All rafts were initiated as circles and simulated until pseudo-steady state treadmilling occurred (wherein *α*≈*δ*). In all simulations, *R* = 0.9 ℓ and *η* = 0.2. A was set to 1.08, 1.42, 1.80, and 3.24 for **S4–S7 Movies**, respectively. Note that a finer separation in A between videos is provided in the range 1.35≤A≤1.59 since this is the approximate range of continuous phase change identified. All scale bars represent 40 mm or ~14 ℓ. The rafts were initiated as circles. Note that these videos are displayed over long durations at approximately 1500x speed. Therefore, the individual transition of agents from one state to another, as well as the motion of freely active agents (red) are not immediately distinguishable.(MP4)

S8 Movie**Modulated Activity** illustrates simulated ant raft comprised of 2,250 agents over a span of 5.5 simulation hours when activity was stepped twice between A=1.1 and A=1.6 to demonstrate how activity can toggle a raft between exploratory and inactive phases of protrusion growth and non-growth, respectively. Instantaneous activity level is indicated at the bottom of the video. The raft was initiated as a circle. *R* = 0.9 ℓ and *η* = 0.2. The scale bar represents 40 mm or ~14 ℓ.(MP4)
