# Supplementary figures and images for: Computational exploration of treadmilling and protrusion growth observed in fire ant rafts
Source: PLoS Comput Biol. 2022 Feb 17;18(2):e1009869. doi: 10.1371/journal.pcbi.1009869 (PMC8890740; doi:10.1371/journal.pcbi.1009869)

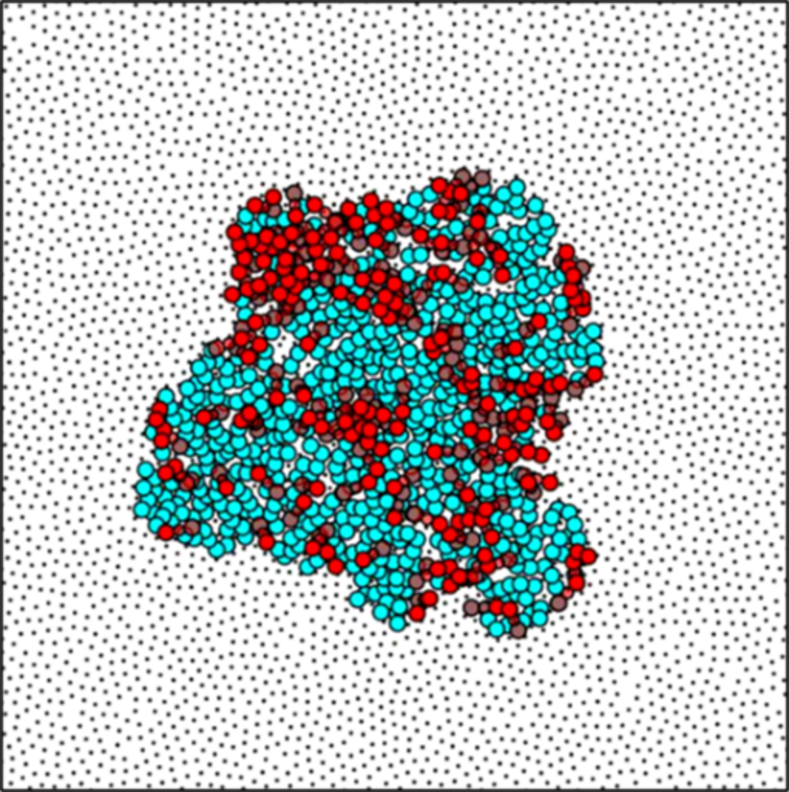

Supplement: S1 Fig — A snapshot of the discrete numerical domain is shown with water nodes plotted as black dots, condensed structural agents plotted as cyan circles, and dispersed freely active agents plotted as red circles. (TIF) [file pcbi.1009869.s001.tif]

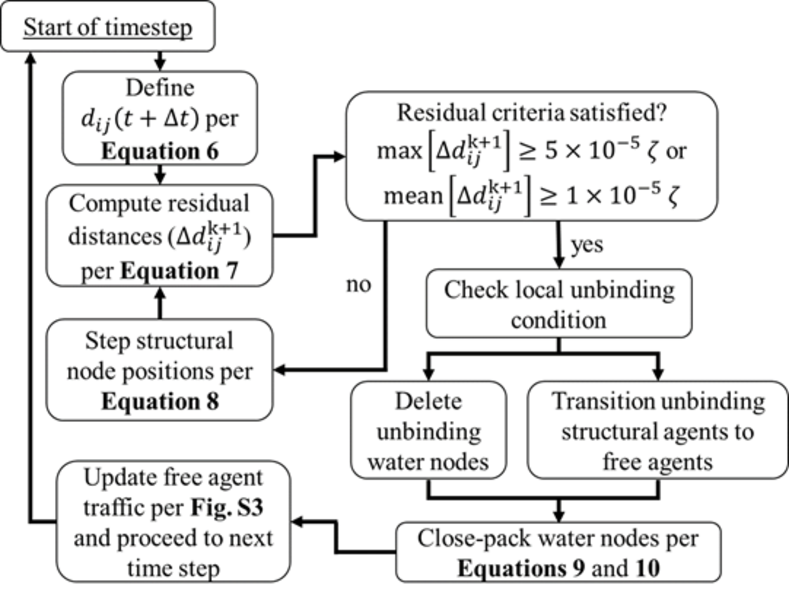

Supplement: S2 Fig — A flow chart details the algorithmic order in which positions of structural agents and water nodes are updated. The point at which unbinding events occur is also displayed. (TIF) [file pcbi.1009869.s002.tif]

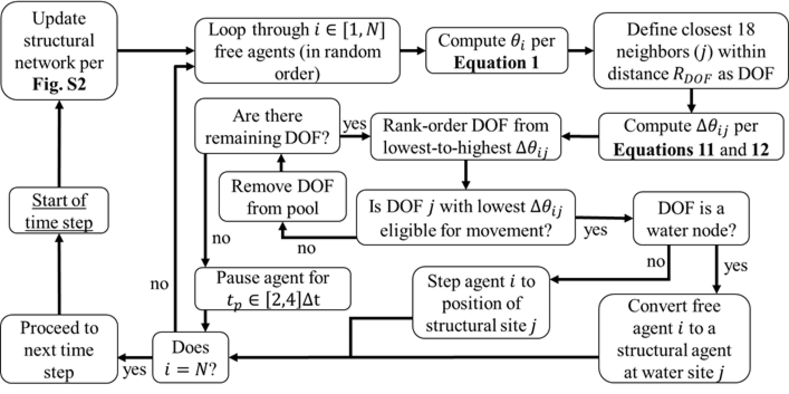

Supplement: S3 Fig — A flow chart details the algorithm by which movement is determined for each freely active agent, in each time step. (TIF) [file pcbi.1009869.s003.tif]

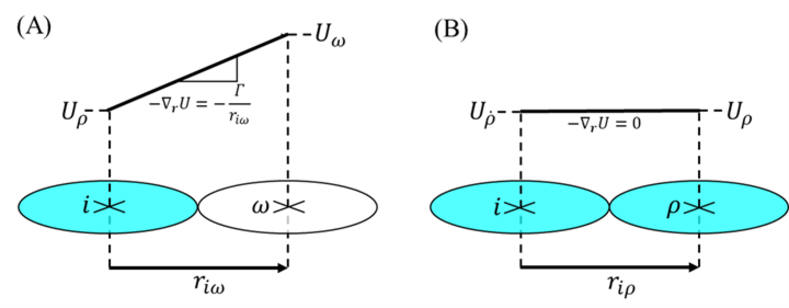

Supplement: S4 Fig — (A) The origins of the pairwise repulsive force acting at the position of structural node i due to the proximity of water node ω is illustrated in 1D. The force is taken as the gradient in energy landscape from ω to i. (B) Similarly, the lack of any pairwise repulsive force acting at the positions between structural node i and structural node ρ is visually illustrated by the lack of a gradient in the local energy. (TIF) [file pcbi.1009869.s004.tif]

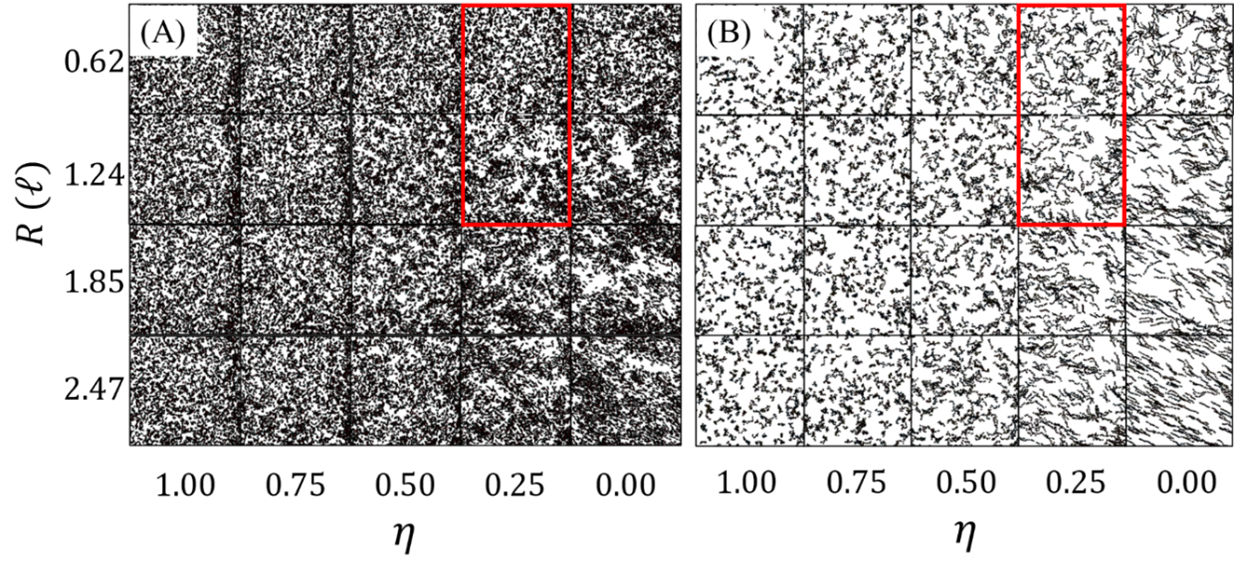

Supplement: S5 Fig — Snapshots of the surface traffic of freely active agents in the numerical model are illustrated at a packing fraction of ϕ = 0.24. The (A) full traffic, as well as (B) streamlines of just 10% of modeled agents composited from 10 time steps, are shown to illustrate the presence of clustering and directional motion, respectively. From top to bottom R is swept over the range R∈[0.62,2.47] ℓ and from left to right, η is swept over the range η = [1.00,0] in increments of 0.25. The regional range that roughly matches experiments is outlined in red for each table. Although the agents’ motions are confined to a lattice of nodes, the lattice is not depicted here for visual clarity. (TIF) [file pcbi.1009869.s005.tif]

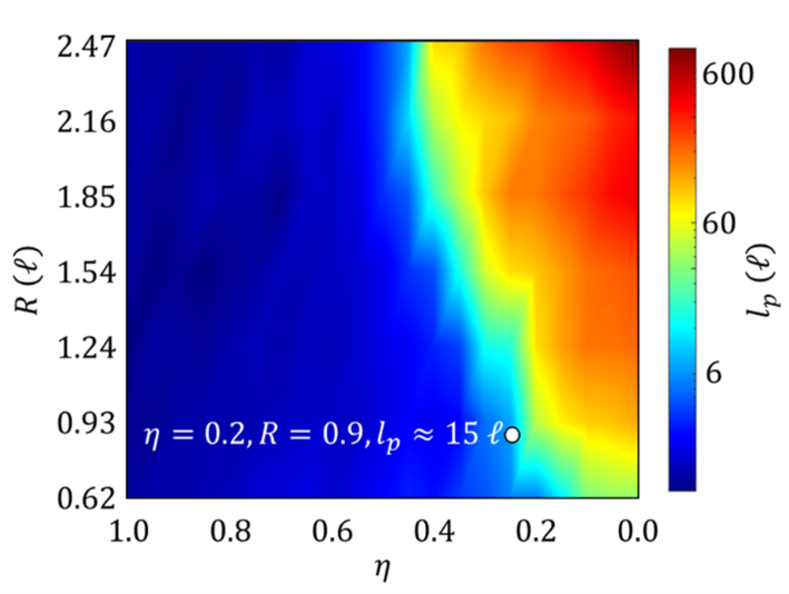

Supplement: S6 Fig — An interpolated, 2D heat map illustrates how lp evolves over the parameter space defined by R∈[0.62,2.47] ℓ and η∈[0,1] in the numerical model. The point that matches the experimental data is plotted as a white dot. (TIF) [file pcbi.1009869.s006.tif]

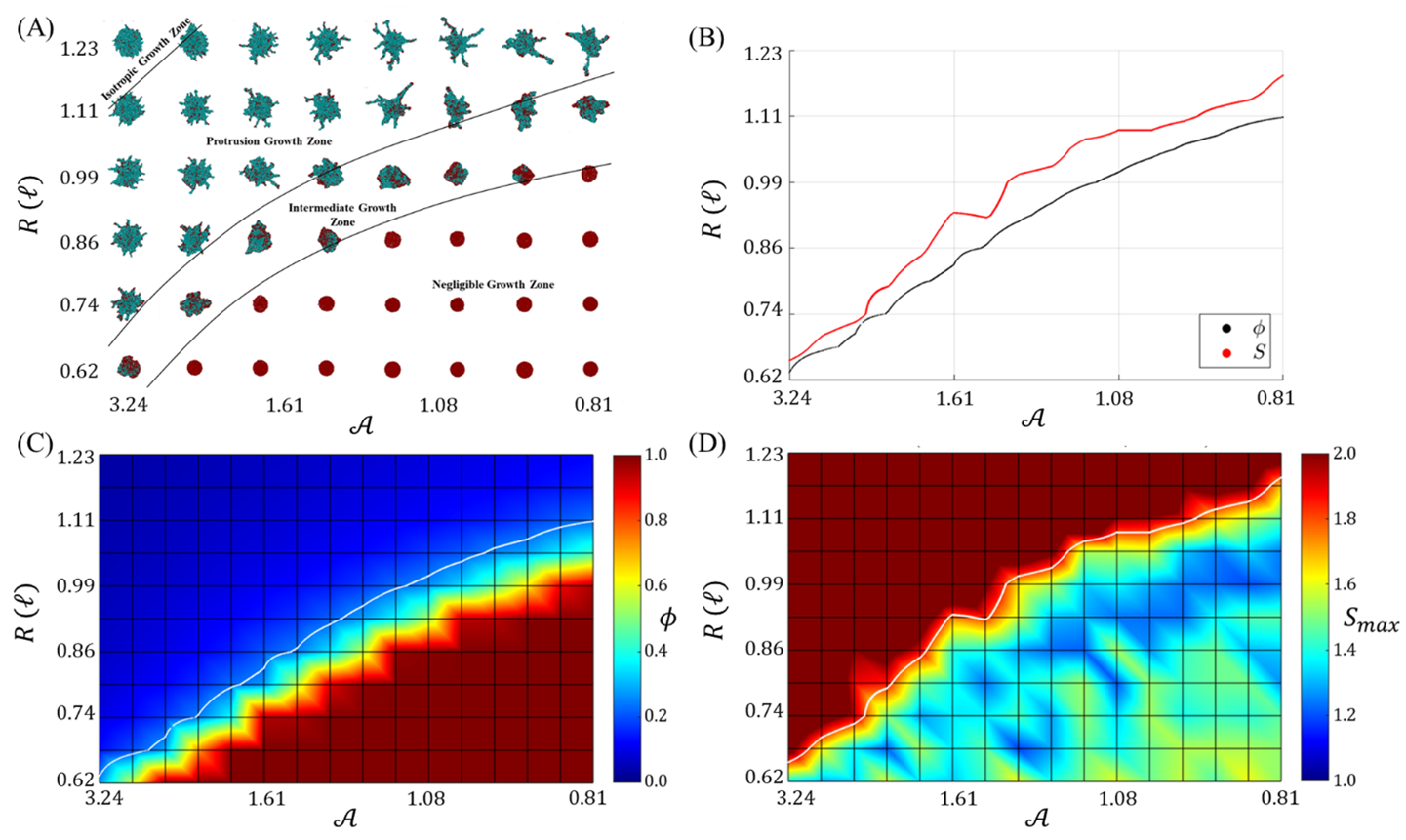

Supplement: S7 Fig — (A) A phase table depicts the morphology of simulated rafts for different values of R and A after approximately 1 hour of simulated time. (B) Interpolated curves with respect to R and A depict the phase space in which the maximum surface excess Smax (red) and packing fraction ϕ (black) matched those of the experiments (~1.8 and ~0.24, respectively) to within 0.25%. (C) A heat map of ϕ with respect to R and A is shown with the white curve corresponding to the black curve from (B). (D) A heat map of Smax is shown with respect to R and A with the white curve corresponding to the red curve from (B). (TIF) [file pcbi.1009869.s007.tif]
